# SARS-CoV-2 variants of concern partially escape humoral but not T cell responses in COVID-19 convalescent donors and vaccine recipients

**DOI:** 10.1126/sciimmunol.abj1750

**Published:** 2021-05-25

**Authors:** Daryl Geers, Marc C. Shamier, Susanne Bogers, Gerco den Hartog, Lennert Gommers, Nella N. Nieuwkoop, Katharina S. Schmitz, Laurine C. Rijsbergen, Jolieke A. T. van Osch, Emma Dijkhuizen, Gaby Smits, Anouskha Comvalius, Djenolan van Mourik, Tom G. Caniels, Marit J. van Gils, Rogier W. Sanders, Bas B. Oude Munnink, Richard Molenkamp, Herbert J. de Jager, Bart L. Haagmans, Rik L. de Swart, Marion P. G. Koopmans, Robert S. van Binnendijk, Rory D. de Vries, Corine H. GeurtsvanKessel

**Affiliations:** 1 Department of Viroscience, Erasmus MC, Rotterdam, Netherlands.; 2 Centre for Immunology of Infectious Diseases and Vaccines, National Institute for Public Health and the Environment, Bilthoven, Netherlands.; 3 Department of Medical Microbiology, Amsterdam UMC, Amsterdam, Netherlands.; 4 Department of Microbiology and Immunology, Weill Medical College of Cornell University, New York, NY 10021, USA.; 5 Department of Occupational Health Services, Erasmus MC, Rotterdam, Netherlands.

## Abstract

The emergence of SARS-CoV-2 variants harboring mutations in the spike (S) protein has raised concern about potential immune escape. Here, we studied humoral and cellular immune responses to wild-type SARS-CoV-2 and the B.1.1.7 and B.1.351 variants of concern in a cohort of 121 BNT162b2 messenger RNA–vaccinated health care workers (HCWs). Twenty-three HCWs recovered from mild COVID-19 disease and exhibited a recall response with high levels of SARS-CoV-2–specific functional antibodies and virus-specific T cells after a single vaccination. Specific immune responses were also detected in seronegative HCWs after one vaccination, but a second dose was required to reach high levels of functional antibodies and cellular immune responses in all individuals. Vaccination-induced antibodies cross-neutralized the variants B.1.1.7 and B.1.351, but the neutralizing capacity and Fc-mediated functionality against B.1.351 were consistently two- to fourfold lower than those against the homologous virus. In addition, peripheral blood mononuclear cells were stimulated with peptide pools spanning the mutated S regions of B.1.1.7 and B.1.351 to detect cross-reactivity of SARS-CoV-2–specific T cells with variants. We observed no differences in CD4^+^ T cell activation in response to variant antigens, indicating that the B.1.1.7 and B.1.351 S proteins do not escape T cell–mediated immunity elicited by the wild-type S protein. In conclusion, this study shows that some variants can partially escape humoral immunity induced by SARS-CoV-2 infection or BNT162b2 vaccination, but S-specific CD4^+^ T cell activation is not affected by the mutations in the B.1.1.7 and B.1.351 variants.

## Teaser

Infection- or vaccination-induced SARS-CoV-2 S–specific CD4^+^ T cells are indifferent to the S mutations of variants of concern.

## INTRODUCTION

The severe acute respiratory syndrome (SARS) outbreak in 2003 was completely contained by nonpharmaceutical interventions, but controlling the spread of SARS coronavirus-2 (SARS-CoV-2) has been more difficult. Countries across the world implemented a large range of social restrictions and measures that differ in stringency and goal (*1*, *2*). A few countries have been successful in interrupting the SARS-CoV-2 transmission chain, but most countries are still facing (multiple) resurgences. Implementation of long-lasting lockdowns is difficult, due to major economic and social disruption, leading to decreased compliance (*3*, *4*). A large part of the world therefore depends on the acquisition of immunity by vaccination, which, in conjunction with public health measures, should contain the coronavirus disease 2019 (COVID-19) pandemic.

It is evident that the fundamental components of the adaptive immune system (B cells, CD4^+^ T cells, and CD8^+^ T cells) contribute to the control of SARS-CoV-2 infection (*5*–*11*). The exact correlates of protection remain to be elucidated (*12*, *13*), but circulating antibodies and memory immune cells are crucial in protection against COVID-19. Especially important are virus-specific neutralizing antibodies targeting the receptor binding domain (RBD) of the spike (S) protein, which correlate with presence of SARS-CoV-2–specific CD4^+^ circulating follicular helper T cells (cT_FH_) (*8*, *14*) and can prevent the interaction between virus and the host cell (*15*). If SARS-CoV-2 establishes a reinfection, memory B and T cells rapidly proliferate and control the infection. Similarly, so-called nonneutralizing antibodies may contribute to clearance via Fc-receptor–mediated killing of virus-infected cells, a process known as antibody-dependent cellular cytotoxicity (ADCC), although this has only been shown in a limited number of studies for COVID-19 (*16*).

Immunological memory is established by an initial priming of the immune system, either by natural infection or by vaccination. SARS-CoV-2 infections may induce lasting immunological memory, although the different components of the adaptive immune system exhibit distinct kinetics. Levels of S-specific immunoglobulin G (IgG) antibodies and virus-specific memory T cells slightly decrease over time, but levels of virus-specific memory B cells increase over the first period of 6 months (*17*–*23*). COVID-19 vaccines were developed at an unprecedented speed and shown to be safe and highly effective in preventing symptomatic SARS-CoV-2 infections (*24*–*27*). Exact kinetics of virus-specific immune responses induced by vaccination remain to be elucidated. Initial results indicate that S-specific binding and neutralizing antibodies slightly decline over a period of several months, although they remain detectable (*28*). Extensive characterization of the cellular immune response to vaccination and its durability is currently ongoing.

Symptomatic SARS-CoV-2 reinfections or breakthrough infections in previously infected or vaccinated individuals occur, but their frequency is unknown and full evidence of reinfection is rarely provided (*29*–*31*). In the phase 3 vaccination trials, almost all breakthrough infections led to mild disease, implicating that partial vaccine-induced immunity still offered protection from severe disease (*24*–*27*). However, the emergence of SARS-CoV-2 variants of concern (VOCs) poses a threat. Divergent strains with an accumulation of mutations in the different S domains are potentially capable of evading infection or vaccination-induced neutralizing antibodies (*32*). These VOCs include the B.1.1.7 lineage that was initially detected in the United Kingdom and has now spread worldwide (*33*), as well as the B.1.351 and P.1 lineages, which were detected in South Africa and Brazil, respectively (*34*). These variants have a number of mutations and deletions compared with previously circulating viruses, some of which are located in the RBD. The B.1.1.7 variant acquired a substitution at amino acid 501 (N501Y), and the B.1.351 and P.1 variants additionally accumulated amino acid substitutions at positions 417 and 484 (K417N/T and E484K). Furthermore, multiple substitutions have independently evolved in the N-terminal domain (NTD) of these variants, suggesting an in vivo selective pressure on the RBD and NTD sites (*32*).

The emergence of VOCs with the reduced susceptibility to polyclonal antibody responses could lead to a growing number of reinfections or breakthrough infections. In Brazil, a COVID-19 resurgence has been reported despite high seroprevalence, partially attributed to circulating strains from the P.1 and P.2 lineages (*35*, *36*). Similarly, reinfections with B.1.1.7 and B.1.351 viruses are being reported (*37*, *38*). Studies into vaccine efficacy against VOCs are crucial and currently ongoing because there is a specific concern regarding efficacy against B.1.351 and P.1. For example, the vaccination efficacy of AZD1222, which was reported to be 70% in the United Kingdom and Brazil, only reached 22% in South Africa (*39*). Reduced efficacy against B.1.351 was also reported for the NVX-CoV237 and Ad26.COV2-S vaccines by the manufacturers Novavax and Johnson & Johnson, respectively (*12*).

Although several studies have demonstrated that some VOCs may be capable of evading infection or vaccination-induced neutralizing antibodies, little is known about T cell cross-reactivity with VOCs. Here, we obtained serum and peripheral blood mononuclear cells (PBMCs) from BNT162b2 mRNA–vaccinated health care workers (HCWs) and assessed humoral and cellular immune responses to wild-type (WT) SARS-CoV-2 and the B.1.1.7 and B.1.351 VOC. HCWs who previously experienced COVID-19 exhibited a rapid and strong recall response upon a single vaccination, whereas seronegative HCWs required two vaccinations to reach comparable levels of humoral and cellular immune responses. The B.1.351 variant was consistently less well recognized and neutralized on the antibody level, and a single vaccination in previously COVID-19–negative donors did not lead to cross-reactive neutralizing antibodies in most of the participants. No differences in CD4^+^ T cell responses against WT, B.1.1.7, and B.1.351 S proteins were detected.

## RESULTS

### COVID-19–naive and –recovered vaccination cohort

**Fig. 1.**
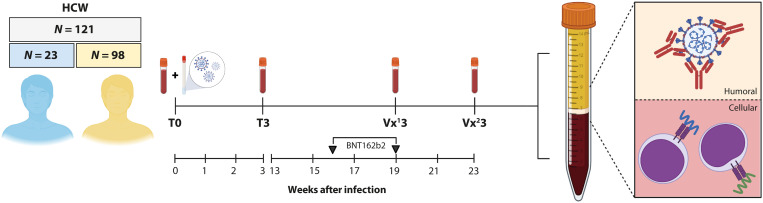
HCW study design. *N* = 121 HCWs were enrolled in a prospective SARS-CoV-2 infection and vaccination study. Upon symptomatic presentation to occupational health services, a paired nasopharyngeal swab and EDTA blood sample was obtained (T0). A second EDTA blood sample was obtained 3 weeks after diagnostic RT-PCR (T3). On the basis of the diagnostic RT-PCR result at T0 and serology result at T3, 98 COVID-19–naive (yellow) and 23 COVID-19–recovered (blue) HCWs were enrolled in the vaccination study on average 50 days after inclusion. *N* = 13 COVID-19–recovered and *N* = 12 COVID-19–naive participants were randomly selected for in-depth analysis. Blood samples were collected after the first (Vx^1^3) and second (Vx^2^3) vaccination, processed, and subsequently used for downstream serological and cellular assays.

From April 2020 onward, HCWs were enrolled in a prospective cohort study upon symptomatic presentation to the occupational health services. Samples were obtained early after onset of COVID-19 symptoms (acute, T0) and 3 weeks later (convalescent, T3). On the basis of results from the diagnostic reverse transcription polymerase chain reaction (RT-PCR) at T0 and serological screening for S-specific antibodies at T3, study participants were classified as COVID-19–naive or –recovered participants. None of the participants that tested positive for COVID-19 were infected with a variant virus harboring the N501Y mutation, and none required hospitalization. From January 2021 onward, *N* = 121 HCWs were included in a prospective vaccination study. The median age of study participants was 41 years, and 9.1% were older than 60 years; 68.9% were female. The median number of days between diagnosis (T0) and administration of the first vaccine dose was 54 days (range, 23 to 232 days). All participants received two doses of the BNT162b2 mRNA vaccine (Pfizer/BioNTech) with an interval of 3 weeks. Among the participants, 19% (*N* = 23) were classified as recovered from prior COVID-19. The study design is shown in Fig. 1, and participant characteristics are summarized in Table 1. Binding antibody assays were performed on samples from all 121 participants, whereas in-depth immunological analyses were performed on a selection of 25 participants (*N* = 13 COVID-19 recovered and *N* = 12 COVID-19 naive). The selection of participants for in-depth analysis was based on availability of longitudinal PBMC samples.

**Table 1.** Characteristics of study participants before vaccination.

	**All**	**COVID-19 recovered**	**COVID-19 naive**	**In-depth analysis**	
				**COVID-19 recovered**	**COVID-19 naive**
***N***	121	23	98	13	12
**Gender**					
Male	39 (32.2%)	6 (26.1%)	33 (35.6%)	3 (23%)	1 (8.3%)
Female	82 (67.8%)	17 (73.9%)	65 (66.3%)	10 (77%)	11 (91.7%)
**Age**	40	42	38.5	42	47
(median + Q1–Q3)	(34.8–55.8)	(34.5–56.5)	(34.3–57.3)	(34.5–56.5)	(34.3–55.0)
<30	21 (17.4%)	4 (17.4%)	17 (17.3%)	3 (23.1%)	1 (8.3%)
30–44	56 (46.3%)	10 (43.5%)	46 (46.9%)	5 (38.5%)	5 (41.7%)
45–59	35 (28.9%)	7 (30.4%)	28 (28.6%)	4 (30.8%)	5 (41.7%)
>60	9 (7.4%)	2 (8.7%)	7 (7.1%)	1 (7.7%)	1 (8.3%)
**Days between diagnosis**					
**and vaccination** (median)	-	54	-	47	-
<30 days		2 (8.7%)		2 (15.4%)	
30–60 days		12 (52.2%)		10 (85.6%)	
>60 days		9 (39.1%)		1 (7.7%)	

### Rapid boosting of S-specific antibodies in COVID-19–recovered donors

**Fig. 2.**
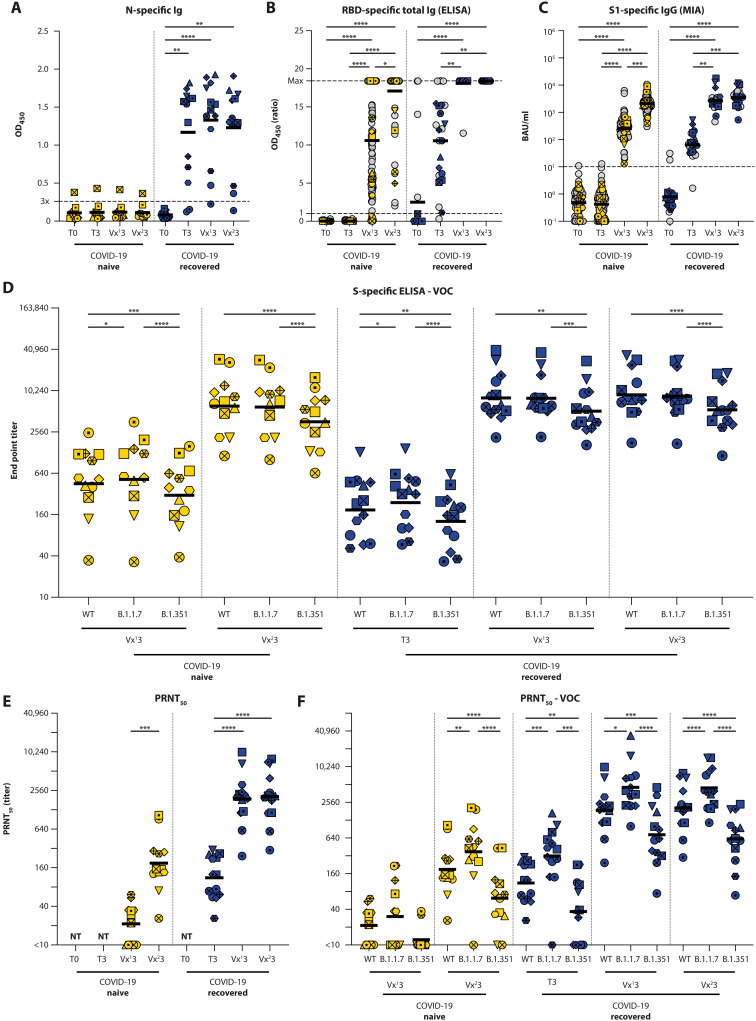
Detection of SARS-CoV-2–specific humoral responses. Total Ig levels were measured in COVID-19–naive (yellow) and –recovered (blue) donors at the acute, convalescent, post-vaccination 1, and post-vaccination 2 stage (T0, T3, Vx^1^3, and Vx^2^3) by an (**A**) ELISA against nucleocapsid (N) and (**B**) RBD. (**C**) Quantitative IgG against S1 was measured by a Luminex bead assay. (**D**) Antibody binding to WT SARS-CoV-2 and VOC B.1.1.7 and B.1.351 was determined by end point titration in ELISA. Virus neutralization was measured by PRNT_50_ against (**E**) WT SARS-CoV-2 (D614G) and (**F**) VOC. Analyses in (B) and (C) were performed on 121 participants, and in-depth analyses were performed in (A), (D), (E), and (F) on 25 participants. Time points in (A), (B), and (C) were compared by performing a nonparametric repeated measures Friedman test. End point titers between VOC in (D) were compared by RM one-way ANOVA or Friedman test. PRNT_50_ titers in (D) and (E) were compared by RM one-way ANOVA. *P < 0.05, **P < 0.01, ***P < 0.001, ****P < 0.0001. Symbol shapes indicate individual donors and are consistent throughout the figures. Lines in (A) and (B) show the means; lines in (C), (D), (E), and (F) show geometric means. Dotted lines represent cutoff values for positivity [3× background OD_450_ in (A), OD_450_ ratio = 1 in (B), and 10.08 BAU/ml in (C)]. NT: not tested.

To confirm previous SARS-CoV-2 infection, sera from the participants selected for in-depth analysis were evaluated for the presence of anti-nucleocapsid (N) Ig antibodies during the acute and convalescent phase, and after the first (μ = 20.3 days and SD = 3.2 days) and second vaccine dose (μ = 26.5 days and SD = 5.9 days) (Fig. 2A and table S1). N-specific antibodies were not detected in 11 of 12 COVID-19–naive donors (1 low positive), whereas substantial levels of N-specific antibodies were detected in 12 of 13 COVID-19–recovered donors. As expected, N-specific antibodies were not boosted by vaccination.

Next, the presence of anti-RBD Ig and anti-S1 IgG antibodies was determined by Wantai enzyme-linked immunosorbent assay (ELISA) and Luminex bead assay [microsphere immunoassay (MIA)] (Fig. 2, B and C, and table S1). The absence of S-specific antibodies before vaccination was confirmed by both assays in the COVID-19–naive cohort, whereas S-specific antibodies were detected before vaccination in 22 of 23 COVID-19–recovered donors [optical density (OD) ratio >1 in Wantai ELISA and binding antibody units (BAU)/ml >10.08 in MIA]. In some participants, S-specific antibodies were already detectable at the time point of symptomatic testing for COVID-19 (T0). Donors selected for in-depth analysis were a good reflection of the total cohort, visualized by the color-coded symbols in Fig. 2 (B and C). No significant differences were observed in binding antibody data as determined by MIA between the total cohort and samples selected for in-depth analysis (table S2).

After one vaccination, all COVID-19–recovered participants showed a surge in antibody levels with OD ratios >10 detected by ELISA (Fig. 2B, *P* < 0.0001, Friedman test). This increase was confirmed by MIA (Fig. 2C, *P* = 0.0001, Friedman test; table S1). The quantitatively interpretable MIA also showed that a second vaccination of COVID-19–recovered participants did not further boost S1-specific IgG antibodies; a plateau was reached after a single shot. In COVID-19–naive participants, 92.5% had detectable total anti-RBD Ig after one vaccine dose, but only 53.2% had an OD ratio >10 (Fig. 2B). In MIA, all participants had detectable antibodies after one vaccination (Fig. 2C and table S1). A clear boosting effect after the second vaccination was observed in COVID-19–naive donors. All participants then had detectable antibodies, and 93.3% had a ratio >10 in ELISA, and significantly higher levels of S1-specific IgG were observed in MIA (geometric mean 252.6 ± 49.55 to 2088.5 ± 287.5 BAU/ml, *P* < 0.0001, Friedman test).

### S-specific antibodies have reduced binding affinity for the B.1.351 S protein

Sera selected for in-depth analysis were initially assessed for their capacity to bind to WT (Wuhan Hu-1), B.1.1.7, and B.1.351 S proteins by ELISA at time points where binding antibodies were detected (post-vaccinations 1 and 2, convalescent sera additionally evaluated for COVID-19–recovered participants) (Fig. 2D). In both COVID-19–naive and –recovered donors and at all time points assessed, sera had reduced binding affinity for the B.1.351 S protein when compared with the WT S protein. In addition, at two time points, a slightly increased binding affinity for B.1.1.7 S was observed (Vx^1^3 for naive donors and T3 for convalescent donors).

### Neutralizing antibodies have reduced activity against B.1.351

Sera selected for in-depth analysis were subsequently tested for the presence of neutralizing antibodies by an infectious virus plaque reduction neutralization test (PRNT_50_) at time points when binding antibodies were detected by ELISA (post-vaccinations 1 and 2, convalescent phase additionally evaluated for COVID-19–recovered participants) (Fig. 2E, individual S-curves in fig. S1). In COVID-19–naive HCWs, a single vaccine dose led to detectable levels of neutralizing antibodies in 7 of 12 donors. PRNT_50_ values were boosted by a second vaccination to detectable levels in all donors (*P* = 0.0005, Wilcoxon test; table S1), but the peak titer was significantly lower compared with COVID-19–recovered participants (geometric mean titer 1:189, *P* < 0.0001, unpaired *t* test). Neutralizing antibodies against WT SARS-CoV-2 (which contained the D614G mutation) were detected in all sera collected from *N* = 13 COVID-19–recovered HCWs before vaccination. A single vaccination boosted the PRNT_50_ titers to a geometric mean plateau value of 1:1874 [*P* < 0.0001, repeated measures (RM) one-way analysis of variance (ANOVA)]. Titers did not increase further after a second vaccination.

Next, cross-reactivity of neutralizing antibodies induced by vaccination or infection against VOCs was evaluated (Fig. 2F). In prevaccination sera from COVID-19–recovered donors, neutralizing antibodies against WT SARS-CoV-2 (D614G), B.1.1.7, and B.1.351 were detected in 13 of 13, 12 of 13, and 7 of 13 donors, respectively (table S1). A single vaccination was sufficient to boost neutralizing antibodies to detectable levels for all SARS-CoV-2 variants, and a second dose did not further boost antibody titers. Compared with PRNT_50_ titers against WT SARS-CoV-2, geometric mean titers against B.1.1.7 were consistently higher [2.5-fold and 2.2-fold increase post-vaccinations 1 and 2, *P* = 0.0114 and *P* < 0.0001 (RM one-way ANOVA), respectively], whereas titers against B.1.351 were consistently lower [2.7-fold and 3.3-fold decrease post-vaccinations 1 and 2, *P* = 0.0004 and *P* < 0.0001 (RM one-way ANOVA), respectively] (summarized in table S3). Neutralizing antibodies were detected after one vaccination in 7 of 12, 6 of 12, and 2 of 12 COVID-19–naive donors against WT SARS-CoV-2, B.1.1.7, and B.1.351, respectively. A second vaccine dose boosted that to 12 of 12, 11 of 12, and 10 of 12. Geometric mean titers after the first vaccination against all VOC were not significantly different, but after the second vaccination, a 2-fold increase of neutralizing antibodies against B.1.1.7 and a 3.1-fold decrease against B.1.351 was detected (*P* = 0.0013 and *P* < 0.0001, respectively, RM one-way ANOVA) (summarized in table S3).

### Antibodies have reduced Fc-mediated functionality against B.1.351

**Fig. 3.**
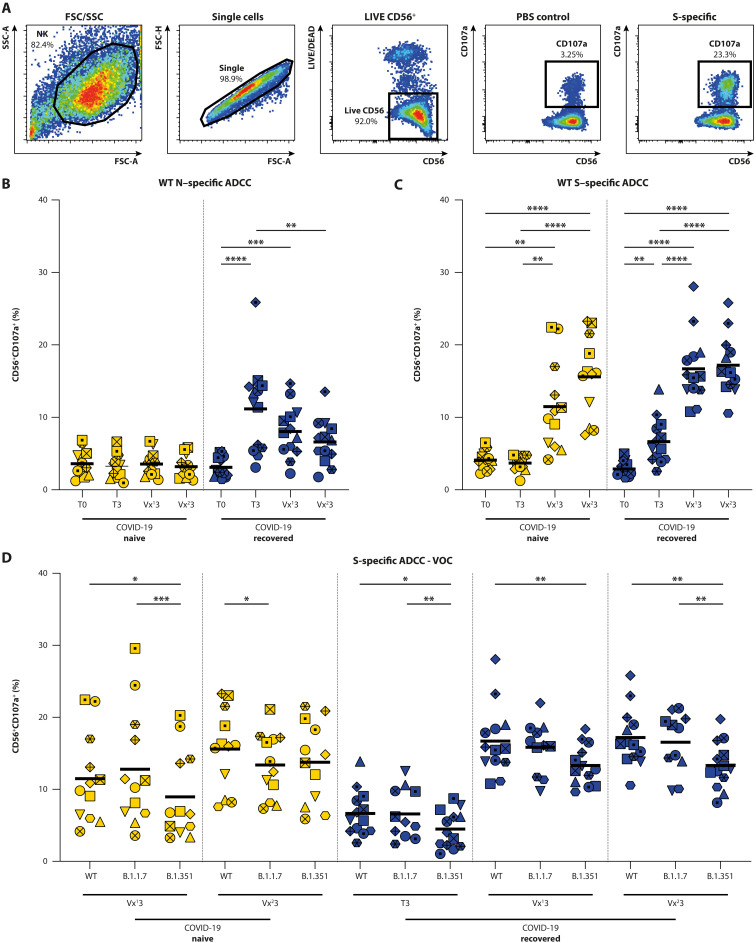
Detection of ADCC-mediating antibodies by measuring NK92.05 degranulation. (**A**) Gating strategy for detection of degranulating NK cells: (i) NK92.05-CD16 cells are selected on the basis of size and granularity, (ii) exclusion of doublets, and (iii) selection of LIVE and CD56^+^ cells. Degranulation is measured as percentage CD107a^+^ cells within the NK fraction; PBS coating is included as background control. (**B** and **C**) ADCC-mediating antibodies were detected in COVID-19–naive (yellow) and –recovered (blue) donors at the acute, convalescent, post-vaccination 1, and post-vaccination 2 stage (T0, T3, Vx^1^3, and Vx^2^3) against the WT N (B) and S (C) protein. (**D**) ADCC-mediating antibody reactivity with WT SARS-CoV-2 and VOC B.1.1.7 and B.1.351. These analyses were performed on 25 participants. Time points in (B) and (C) were compared by performing a nonparametric repeated measures Friedman test. Differences between variants were assessed by mixed-effect models. *P < 0.05, **P < 0.01, ***P < 0.001, and ****P < 0.0001. Symbol shapes indicate individual donors and are consistent throughout the figures. Lines indicate mean responses.

Next, sera were evaluated for the presence of antibodies that could activate natural killer (NK) cells as a proxy for ADCC. A set dilution of serum (1:100) was incubated on plates coated with hexahistidine (His)–tagged proteins [WT N, WT S (Wuhan Hu-1), B.1.1.7 S, and B.1.351 S], followed by addition of an immortalized FcγRIII^+^ NK cell line. Activation of NK cells was measured using flow cytometry by detecting lysosomal-associated membrane protein–1 (LAMP-1 or CD107a^+^; gating strategy shown in Fig. 3A). N-specific ADCC–mediating antibodies were not detected in the *N* = 12 COVID-19–naive donors at any time point (Fig. 3B and table S1). In *N* = 13 COVID-19–recovered donors, N-specific ADCC-mediating antibodies were detected in the convalescent phase initially. These N-specific antibodies gradually waned over time (convalescent versus post-vaccination 2: *P* = 0.005, Friedman test). As expected, vaccination did not boost N-specific antibodies.

In COVID-19–naive donors, WT S–specific ADCC-mediating antibodies were not detected before vaccination, whereas in COVID-19–recovered donors, WT S–specific ADCC-mediating antibodies were detected in the convalescent phase (Fig. 3C, *P* = 0.0038, RM one-way ANOVA; table S1). In COVID-19–naive donors, the first vaccination led to low-level detection of ADCC-mediating antibodies, which were further boosted by the second vaccination. In COVID-19–recovered donors, ADCC-mediating antibodies were already boosted by a single vaccination to peak levels, and the second shot did not lead to an additional boosting (reminiscent of binding and neutralizing antibodies; see Fig. 2).

Percentages of degranulating NK cells were comparable between WT and B.1.1.7 S at all time points, regardless of whether donors had been previously exposed to SARS-CoV-2 or not, and the number of vaccinations. However, ADCC-mediating antibodies had significantly reduced activity against B.1.351 S at almost all time points in COVID-19–naive and –recovered donors (tested by mixed-effect models; Fig. 3D). Even in recovered donors after two vaccinations, this reduced activity to B.1.351 was apparent. Percentages of CD107a^+^ NK cells were significantly correlated to the binding antibody titers (shown in Fig. 2D); however, the correlation was not evident at all time points assessed separately (fig. S2).

### Rapid boosting of S-specific T cells in COVID-19–recovered donors

**Fig. 4.**
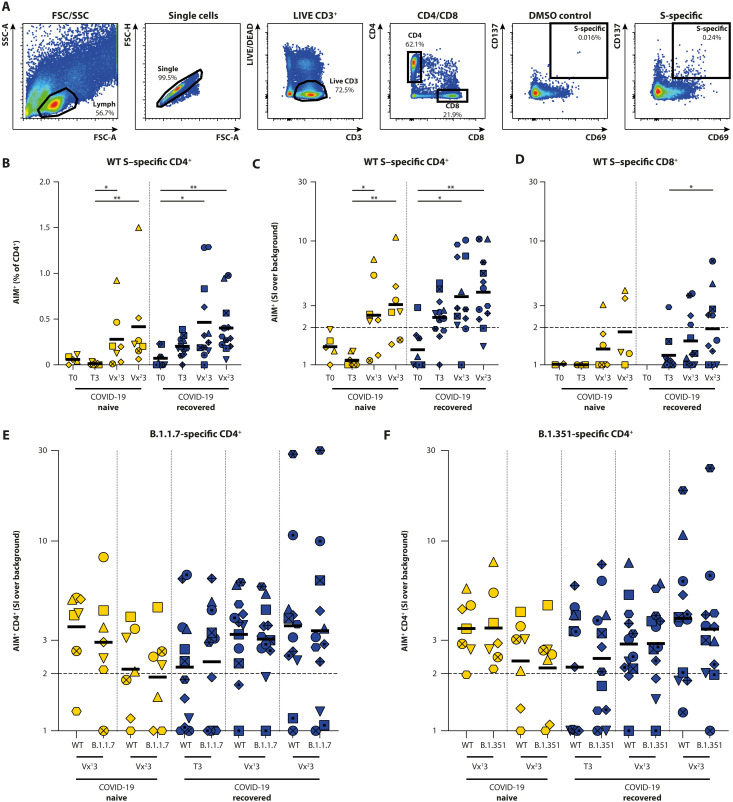
Detection of S-specific T cells by measuring up-regulation of AIM. (**A**) Gating strategy for virus-specific T cells that up-regulate AIM: (i) Lymphocytes are selected on the basis of size and granularity, (ii) exclusion of doublets, (iii) selection of LIVE and CD3^+^ cells, and (iv) division into CD4^+^ and CD8^+^ T cells. Activation is measured as percentage CD69^+^/CD137^+^ double-positive cells within the CD4 or CD8 fraction; DMSO stimulation is included as background control. (**B** to **D**) Antigen-specific activation of CD4^+^ and CD8^+^ T cells in COVID-19–naive (yellow) and COVID-19–recovered (blue) donors at the acute, convalescent, post-vaccination 1, and post-vaccination 2 stage (T0, T3, Vx^1^3, and Vx^2^3) by overlapping peptide pools covering the full WT S protein. Activation of SARS-CoV-2–specific CD4^+^ T cells is shown as percentage of AIM^+^ cells within the CD4^+^ subset after (B) subtraction of the DMSO background or (C) as an SI by dividing specific activation over background activation. (D) Activation of SARS-CoV-2–specific CD8^+^ T cells is shown as SI. An SI of 2 or higher is considered a positive T cell response. (**E** and **F**) Antigen-specific activation of CD4^+^ T cells by peptide pools exclusively covering mutational regions in VOC B.1.1.7 and B.1.351, compared against homologous WT peptide pools. Antigen-specific T cell responses are shown as SI. These analyses were performed in 20 participants. Time points in (B), (C), and (D) were compared by performing a Kruskall-Wallis test. Differences between variants were compared by performing Wilcoxon test. *P < 0.05 and **P < 0.01. Symbol shapes indicate individual donors and are consistent throughout the figures. Lines indicate mean (B) or geometric mean (C to F) responses. Low cell count samples (<10,000 or <5000 events within CD4^+^ or CD8^+^ gate, respectively) were excluded.

Besides serological responses, we assessed the presence of S-specific T cell responses in COVID-19–naive (*N* = 7) and –recovered (*N* = 13) HCWs in the acute and convalescent phase and after vaccination. To this end, PBMCs were stimulated with either overlapping peptide pools representing the full-length WT S protein (Wuhan Hu-1) or peptide pools covering the selected mutated regions in the S protein from the B.1.1.7 and B.1.351 VOC. After stimulation, activation-induced marker (AIM; CD69 and CD137) expression within CD4^+^ and CD8^+^ subsets was measured by flow cytometry (gating strategy shown in Fig. 4A). Up-regulation of OX40 and CD137 was additionally assessed in the CD4^+^ subset from *N* = 11 donors and correlated significantly to the up-regulation of CD69 and CD137 (fig. S3).

In COVID-19–naive donors, WT S–specific CD4^+^ T cells expressing AIM (CD69^+^CD137^+^) were detected after the first and second vaccination (Fig. 4B, T3 to Vx^1^3, *P* = 0.0374; T3 to Vx^2^3, *P* = 0.0016, Kruskal-Wallis test). In COVID-19–recovered donors, S-specific CD4^+^ T cells were already detected in the convalescent phase, which were boosted after the first vaccination. A second vaccination did not lead to an additional boosting effect (Fig. 4B). Similar results were observed when a stimulation index (SI) was calculated (Fig. 4C, ratio of S-specific CD4^+^ T cell activation over background activation). Previously, we regarded individuals with an SI > 3 as responders; however, this was in an intensive care unit cohort with strong T cell responses (*11*). Because the strength of the T cell response seems to be correlated to disease severity (*40*, *41*), we lowered the arbitrary cutoff to an SI > 2 to identify responders after mild COVID-19 (table S1). In COVID-19–naive donors, CD4^+^ T cell responders were not observed before vaccination, whereas five of seven responders were identified after one or two vaccinations. In COVID-19–recovered donors, 8 of 12 responders were identified before vaccination, which increased to 10 of 12 responders after one or two vaccinations.

CD8^+^ S–specific T cell responses in COVID-19–naive (*N* = 7) and –recovered (*N* = 13) HCWs were more difficult to detect. In COVID-19–naive participants before vaccination, CD8^+^ T cell responses were never observed. A nonsignificant trend for increasing CD8^+^ T cell responses (both AIM and SI, Fig. 4D; data at T0 from COVID-19–recovered donors lacking due to low counts in CD8^+^ gate) was observed after vaccination of COVID-19–negative donors. In COVID-19–recovered participants, CD8^+^ S–specific T cells were already observed in the convalescent phase, with increased levels after two vaccinations (*P* = 0.046, Wilcoxon rank test).

### S-specific CD4^+^ T cells have comparable reactivity with VOCs

Subsequently, we assessed T cell responses to VOCs B.1.1.7 and B.1.351. Instead of using overlapping peptide pools covering the full S protein, we used commercially available peptide pools that specifically covered the mutated regions and compared the responses with the corresponding WT control pools containing homologous peptides derived from the WT strain. With these pools, S-specific CD4^+^ T cell responses were again detected (SI > 2) in the post-vaccination samples from COVID-19–naive samples and in the convalescent and post-vaccination samples of COVID-19–recovered donors (Fig. 4, E and F, shown as percentages of activated cells within the CD4^+^ T cell subset in fig. S4), but T cell responses were not observed before vaccination in the COVID-19–naive participants. In both COVID-19–naive and –recovered participants, no differences were observed between CD4^+^ T cell responses to WT S and B.1.1.7 S (Fig. 4E and fig. S4A), or between WT S and B.1.351 S (Fig. 4F and fig. S4B). In addition, we measured the production of interferon-γ (IFN-γ) in cell culture supernatant in response to WT and mutant pools in a subset of samples and did not observe any significant differences in CD4^+^ T cell reactivity to mutant S peptide pools (fig. S5). Because we observed minimal SARS-CoV-2–specific CD8^+^ T cell responses, we did not draw any conclusions on CD8^+^ T cell responses cross-reactive to VOCs.

## DISCUSSION

Here, we show that in both COVID-19–recovered and –naive individuals, BNT162b2 mRNA vaccination induces robust SARS-CoV-2–specific neutralizing antibodies, ADCC-mediating antibodies, and CD4^+^ and CD8^+^ T cells. In line with previous studies (*42*), a single vaccination led to a rapid and strong recall response in COVID-19–recovered participants, without detectable boosting after the second dose. In COVID-19–naive individuals, a second dose was required to consistently detect neutralizing antibodies. Vaccination-induced antibodies did cross-neutralize the variants B.1.1.7 and B.1.351, but the neutralizing capacity and Fc-mediated functionality against B.1.351 were consistently two- to fourfold lower than those against the homologous virus. Last, we detected SARS-CoV-2–specific CD4^+^ and CD8^+^ T cells after infection and vaccination, and CD4^+^ T cell activation was robust and appeared indifferent to the S mutations of the B.1.1.7 and B.1.351 variants.

The study was performed in a selected population of HCWs working with COVID-19 patients who were eligible for rapid vaccination in the Netherlands. The participants were relatively young, predominantly female, with a history of mild COVID-19 disease if previously infected. The population was underpowered to compare vaccination responses in distinct subsets of participants (e.g., age groups and comorbidity). Future studies are required to analyze responses in these specific populations and, more specifically, in vulnerable populations at risk for a less efficient vaccination response (*43*).

Serological screening assays ideally correlate with the neutralizing capacity of detected SARS-CoV-2–specific antibodies. Although the PRNT_50_ with infectious virus is considered the gold standard to measure neutralizing antibody titers, its laborious nature at high biosafety containment level makes it an inappropriate tool for testing of large sample sets and surveillance purposes. To detect antibodies in the complete HCW cohort, we have therefore used well-validated high-throughput assays targeting the S1 or RBD regions of the S protein (Wantai total Ig ELISA and Luminex MIA). Although the Wantai ELISA is a qualitative assay, a ratio of >10 has previously been shown to correspond to virus neutralization (*44*). Because this sensitive assay is often used in diagnostic laboratories, we show here that the presence of antibodies in response to vaccination (in the absence or presence of preexisting SARS-CoV-2 immunity) can indeed be measured but not accurately quantified. The Luminex MIA, on the other hand, allowed clear interpretation and quantitative comparison of antibody responses within the groups and at different time points (*45*).

Although seroconversion was observed in all groups after vaccination, the COVID-19–naive group required a second vaccination to obtain neutralizing titers equal to or higher than the cutoff titer of 1:20. Even after the second vaccination, COVID-19–naive participants had lower neutralizing antibody titers compared with COVID-19–recovered participants (after one vaccination). In hospitalized patients, we have previously shown that a serum neutralizing antibody titer of at least 1:20 was independently associated with the absence of shedding of infectious SARS-CoV-2 (*46*). The neutralization data reported here are in line with previous reports (*42*, *47*–*49*).

The B.1.1.7 and B.1.351 variants both contain the signature mutation N501Y, associated with increased affinity for the ACE2 (angiotensin converting enzyme 2) receptor. We ascertained that none of the COVID-19–recovered individuals in our study had been infected by SARS-CoV-2 harboring this N501Y mutation and that, therefore, the observed neutralization against VOCs in this study was based on cross-reactivity and not on previous priming with a B.1.1.7 or B.1.351 virus. Concerning neutralization of VOCs, recent studies have consistently reported lower neutralizing antibody titers against B.1.351 after vaccination but not against B.1.1.7 (*39*, *42*, *48*–*59*). In our study, we detected consistently increased neutralizing antibody titers in polyclonal sera against B.1.1.7 when compared with WT, both after SARS-CoV-2 infection and vaccination. In some sera, we observed increased binding to B.1.1.7 S by ELISA as well, but not at all time points, thus not explaining the increased neutralization. Our study differs from most previously conducted studies because infectious viruses were used to determine neutralizing antibody titers (in contrast to pseudotyped viruses), potentially explaining this observation. We also observed lower serum neutralization efficiency of the B.1.351 VOC, but none of the sera of vaccinated participants in our cohort showed a complete escape of this variant. Future studies are required to evaluate to what extent the differences in neutralizing antibody titers correlate with the risk of breakthrough infections.

In addition to direct virus neutralization, antibodies can have multiple other modes of action that are primarily mediated by IgG1 and IgG3 subclass antibodies (*60*). After binding to antigens displayed on virus-infected cells, the Fc domain of the antibody engages Fc receptors on effector cells that subsequently kill virus-infected cells, a process known as ADCC. ADCC is mainly mediated by the interaction between virus-specific antibodies and Fcγ receptor IIIα (FcγRIIIα, CD16), which is, for example, present on NK cells (*61*–*63*). Little is known about the role of ADCC-mediating antibodies in COVID-19, but NK cells were shown to be decreased in patients with COVID-19 in association with disease severity (*64*). In ex vivo studies, NK cells from patients with COVID-19 displayed impaired cytotoxicity (*64*, *65*), potentially due to cytokine dysregulation and high plasma levels of interleukin-6 and tumor necrosis factor–α (*66*). ADCC-mediating antibodies have been detected in plasma of COVID-19 convalescent donors (*16*, *67*, *68*). We show that S-specific ADCC-mediating antibodies are induced by both infection and vaccination, but we detect reduced NK cell activation in response to binding to the B.1.351 S protein. Although this reduced activation directly correlated to reduced binding of sera to VOC antigens, a significant correlation was often not observed in sera collected upon multiple exposures to the S antigen. Therefore, the quantity of S-binding antibodies does not fully explain the reduced Fc-mediated effector functionality. In addition to measuring NK cell activation by specific antibodies, future studies should measure functional cytotoxicity using the combination of infectious virus and primary cells.

Whereas previous studies have focused on potential immune evasion by VOC on the antibody level, little is known about immune escape at the T cell level. A small study assessed 45 mutations in the B.1.351 S protein, and found that only 1 mutation overlapped with a low-prevalent CD8^+^ T cell epitope (*69*). A study that followed a sequencing-based approach identified mutations in CD8^+^ T cell epitopes after sequencing 747 SARS-CoV-2 isolates and showed that tetramer-sorted CD8^+^ T cell clones responded to mutant peptides in a transcriptionally different manner (*70*). We performed a comprehensive flow cytometry analysis of SARS-CoV-2–specific CD4^+^ and CD8^+^ T cells from COVID-19–naive and –recovered donors before and after mRNA vaccination. In our analyses, S-specific CD4^+^ and CD8^+^ T cells were induced or boosted by the BTN162b2 vaccine, and the induced CD4^+^ T cells equally recognized the WT, B.1.1.7, and B.1.351 S proteins. This is in line with one other recently performed study that additionally showed cross-reactivity to the P.1 and CAL.20C (B.1.429) variants (*71*). Our study on specific T cells has some limitations: (i) The sample size for which in-depth T cell profiling was performed was limited to 20 donors; (ii) we only focused on S-specific T cells; and (iii) we were not able to further address CD8^+^ T cell responses to VOC because of low frequency of SARS-CoV-2–specific CD8^+^ T cells. Although we hypothesize that this was due to minimal disease severity in this HCW cohort (*40*, *41*), alternatively, this could be because of limited sensitivity when using peptide pools containing overlapping 15-nucleotide oligomers. Additional studies with smaller peptides (8- to 10-nucleotide oligomers) predicted or shown to bind human leukocyte antigen class I are advised to specifically study VOC cross-reactive CD8^+^ T cell responses. In some donors, we also observed the presence of SARS-CoV-2 S–specific T cells before exposure to antigen. This is probably due to the detection of seasonal human coronavirus (HCoV)–specific T cells cross-reactive with SARS-CoV-2. Detection of cross-reactive T cells via the AIM assay was previously described (*7*, *11*, *72*). Although verification of our methods with a potentially more discriminative assay like IFN-γ ELISpot (*73*–*75*) could be useful, the sensitive AIM assay may be the best-suited assay to detect small differences in reactivity to different S antigens.

We generated extensive immunological response profiles against WT SARS-CoV-2, and VOCs B.1.1.7 and B.1.351. However, additional VOC and variants of interest (VOIs) are continuously emerging (*76*). We did not analyze immune responses to the P.1 VOC, originating from Brazil, which also contains mutations in the RBD at positions 417 and 484 (in combination with unique mutations throughout the S protein). Although this variant was not investigated in the current study, previous studies have demonstrated a similar, less pronounced, decrease in neutralization by polyclonal sera after BNT162b2 vaccination (*47*, *77*). Little is known about cross-reactivity on the T cell level to this VOC. Future studies should also focus on complete and in-depth immunological profiling of VOCs and VOIs.

In conclusion, this study emphasizes the importance of a complete assessment of functional immune responses to VOCs or VOIs. We confirm that a single mRNA BNT162b2 vaccination is sufficient to induce vigorous immune responses in previously COVID-19–recovered individuals, on both the humoral and cellular level. In addition, we show that polyclonal sera have reduced functionality against VOC B.1.351, but that CD4^+^ T cell activation in response to the S protein of this variant (and VOC B.1.1.7) was robust. On the basis of these results, we hypothesize that the reduced antibody responses to VOC lead to an increased risk of breakthrough infections with these variants, especially when antibody titers wane after vaccination. However, protection against severe disease caused by these VOC may still be provided by cross-reactive SARS-CoV-2–specific T cell–mediated immunity. Future studies are required to assess to what extent sterile immunity is a requirement to reach herd immunity and interrupt SARS-CoV-2 circulation. Continuous surveillance will monitor the incidence of breakthrough infections and duration of vaccine-induced immunity.

## MATERIALS AND METHODS

### Study design

The Erasmus MC COVID-19 HCWs study was approved by the institutional review board (medical ethical committee, MEC-2020-0264). Informed consent was obtained from all participants. At an early stage in the COVID-19 pandemic, *N* = 121 symptomatic HCWs presenting to the occupational health services from Erasmus MC, Rotterdam, the Netherlands, were enrolled into a prospective cohort study. Serum samples were obtained from all participants during the acute phase (T0, time of diagnostic RT-PCR) and the convalescent phase (T3, 3 weeks after RT-PCR); additional PBMCs were obtained from *N* = 20 participants. On the basis of the diagnostic RT-PCR result at T0 and serology result at T3, study participants were classified as COVID-19–naive (*N* = 98) or –recovered (*n* = 23) participants. As soon as vaccines became available for selected groups of HCWs, starting in January 2021, study participants were enrolled into a vaccination study. Participants received two doses of the BNT162b2 mRNA vaccine with an interval of 21 days. Follow-up samples were collected 14 to 21 days after the first dose and 21 to 28 days after the second dose. Study design is also shown in Fig. 1.

### RT-PCR assays for the detection of single-nucleotide polymorphisms associated with SARS-CoV-2 VOC

The deletion in spike protein (HV69-70) that is indicative of the B.1.1.7 variant was detected by the Applied Biosystems TaqPath COVID-19 kit (Thermo Fisher Scientific). The assay was performed according to the instructions of the manufacturer. This COVID-19 detection kit combines RT-PCR on the Orf1ab, *N* and *S* genes. Failure of detection of the *S* gene in combination with detection of SARS-CoV-2 RNA for the Orf1ab and *N* genes is indicative for the HV69-70 deletion in spike (*S* gene target failure) (*78*). In addition, the presence of the asparagine-to-tyrosine mutation at spike protein position 501 (N501Y) was detected by the VirSNiP N501Y assay (TIB Molbiol A23063T) according to the instructions of the manufacturer. The combination of delHV69-70 and N501Y was considered to be indicative for B1.1.7. Detection of N501Y alone was considered to be indicative for B.1.351.

### PBMCs and plasma isolation

Serum was collected in 10-ml tubes without anticoagulant, centrifuged at 2500 rpm for 15 min, aliquoted, and stored at −20°C for further experiments. PBMCs were isolated from blood collected in K_3_EDTA tubes by density gradient centrifugation. Briefly, blood collection tubes were centrifugated at 2500 rpm for 15 min, after which plasma was aliquoted and stored at −20°C. The cellular fraction was diluted in phosphate-buffered saline (PBS), layered on a density gradient (Lymphoprep, STEMCELL Technologies), and PBMCs were separated by centrifugating at 2000 rpm for 30 min. PBMCs were washed four times in PBS and frozen in 90% fetal bovine serum (FBS) with 10% dimethyl sulfoxide (DMSO; Honeywell) at −135°C until use in stimulation assays.

### RBD/Wantai and N-specific antibody ELISA

Humoral immune responses to vaccination were analyzed using a qualitative ELISA for the detection of total antibodies against the SARS-CoV-2 RBD (Beijing Wantai Biological Pharmacy Enterprise Co. Ltd.) as described previously (*44*). OD ratios above 1.0 were interpreted as positive as indicated by the manufacturer. In this assay, ratios above 10 are indicative of the presence of neutralizing antibodies.

Antibody levels against nucleocapsid (N) and spike (S) in serum were measured by performing an in-house–developed ELISA. ELISA plates were coated with a His-tagged N or S protein (25 or 20 ng per well, respectively) at 4°C overnight. After coating, plates were blocked, washed, and incubated with a dilution series of plasma (1:40 to 1:2560 for N, data from 1:40 used in Fig. 2A; 1:20 to 1:163,840 for S) at 37°C for 2 hours, after which plates were washed and horseradish peroxidase–labeled rabbit anti-human IgG (1:6000; Dako) was added. Plates were incubated at 37°C for 1 hour, washed, and developed by using 3,3′,5,5′-tetramethylbenzidine (KPL). Plates were measured at an OD of 450 nm (OD_450_) using an ELISA microtiter plate reader. OD_450_ signal was corrected by subtracting background signal in the OD_620_ channel. The cutoff was set at 3× average background of multiple negative control sera. For the S ELISA, an arbitrary cutoff value was set at OD_450_ = 0.300, which was used to calculate an end point titer.

### Luminex bead assay

Serum samples were tested for the presence of IgG antibodies to SARS-CoV-2 S1 using a previously published fluorescent bead–based immune assay (*45*). The specificity (99.7%) and sensitivity (91.6%) of the assay were determined using a heterogeneous sample set including asymptomatic and mild to severe COVID-19 cases as representative of COVID-19 cases in the general population, as well prepandemic population samples and samples of persons infected with various viruses, including endemic coronaviruses, as negative controls (*23*). Concentrations were interpolated from a reference consisting of pooled sera using a five-parameter logistic fit and the National Institute for Biological Standards and Control/World Health Organization COVID-19 reference serum 20/136 and expressed as international BAU/ml. A BAU/ml value of >10.08 was considered positive.

### PRN assay

We used the PRNT in which we tested serum samples for their neutralization capacity against SARS-CoV-2 variants as previously described (*9*). Viruses used in the assay were isolated from diagnostic specimen at the Department of Viroscience, Erasmus MC, cultured, and subsequently sequenced to rule out additional mutations in the S protein: D614G [Global Initiative on Sharing All Influenza Data (GISAID): hCov-19/Netherlands/ZH-EMC-2498], B.1.1.7 (GISAID: hCov-19/Netherlands/ZH-EMC-1148), and B.1.351 (GISAID: hCov-19/Netherlands/ZH-EMC-1461). Heat-inactivated sera were twofold diluted in Dulbecco’s modified Eagle’s medium (DMEM) supplemented with NaHCO_3_, Hepes buffer, penicillin, streptomycin, and 1% FBS, starting at a dilution of 1:10 in 60 μl. We then added 60 μl of virus suspension to each well and incubated at 37°C for 1 hour (leading to ±1000 plaques per well in infection controls). After 1 hour of incubation, we transferred the mixtures on to Vero-E6 cells and incubated for 8 hours. After incubation, we fixed the cells with 10% formaldehyde and stained the cells with polyclonal rabbit anti–SARS-CoV-2 nucleocapsid antibody (Sino Biological) and a secondary peroxidase-labeled goat anti-rabbit IgG (Dako). We developed signal by using a precipitate-forming 3,3′,5,5′-tetramethylbenzidine substrate (TrueBlue; Kirkegaard & Perry Laboratories) and counted the number of infected cells per well by using an ImmunoSpot Image Analyzer (CTL Europe GmbH). The dilution that would yield 50% reduction of plaques (PRNT_50_) compared with the infection control (included on all plates) was estimated by determining the proportionate distance between two dilutions from which an end point titer was calculated. We considered a titer >20 to be positive based on assay validation.

### PBMCs and NK92.05 cell culture

Experiments with PBMCs were performed in Gibco Roswell Park Memorial Institute 1640 medium (Gibco) supplemented with 10% human serum (Sanquin, Rotterdam), penicillin (100 IU/ml; Lonza, Belgium), streptomycin (100 μg/ml; Lonza, Belgium), and 2 mM l-glutamine (Lonza, Belgium; R10H medium). Antibody-dependent cell-mediated cytotoxicity experiments were performed with the FcγRIII (CD16)–expressing NK92.05 NK cell line (NK92.05-CD16). The NK92.05-CD16 continuous cell line was from K. S. Campbell at the Fox Chase Cancer Center in Pennsylvania (*79*). NK92.05-CD16 cells were cultured in sterile filtered α-MEM supplemented with sodium bicarbonate [2.2 g/liter, (pH 7.2)], 2-mercaptoethanol (0.0001 M), l-glutamine (200 mM; Gibco), myo-inositol (0.2 mM), 10% horse serum, 10% FBS, folic acid (0.004 mM), sodium pyruvate (1 mM), penicillin (100 IU/ml), and streptomycin (100 μg/ml). Cells were cultured in the presence of human recombinant IL-2 (100 IU/ml). CD16 expression in this cell line was monitored by flow cytometry by staining with anti–CD16-AF647 (clone 3G8; Southern Biotech, 1:50).

### ADCC assay

The presence of ADCC-mediating antibodies in plasma was measured using soluble prefusion stabilized His-tagged S proteins. The constructs contained the following mutations compared with the WT variant (Wuhan Hu-1; GenBank: MN908947.3): deletion (Δ) of H69, V70 and Y144, N501Y, A570D, D614G, P681H, T716I, S982A, and D1118H in B.1.1.7; and L18F, D80A, D215G, L242H, K417N, E484K, N501Y, D614G, and A701V in B.1.351. All S constructs were cloned into a pPPI4 expression vector containing a His tag, verified by Sanger sequencing and subsequently produced in human embryonic kidney–293F cells, and purified as previously described (*6*, *80*). ADCC was also assessed against SARS-CoV-2 His-tagged full-length N (Sino Biological). ADCC assay was performed as previously described (*61*). In short, high-binding 96-well flat bottom plates (Immunolon) were coated with 200 ng of SARS-CoV-2 S or N per well, incubated at 4°C overnight, blocked in 5% bovine serum albumin, washed with PBS, and incubated with plasma (1:100) at 37°C for 2 hours. Plates were washed in PBS, and 100,000 NK92.05-CD16 cells were added in combination with anti–CD107a-V450 (1:100, clone H4A3; BD), GolgiStop (0.67 μl/ml; BD), and golgiplug (1 μl/ml; BD). Plates were incubated at 37°C for 5 hours. Cells were subsequently stained with anti–CD56-PE (clone B159; BD, 1:25), and LIVE/DEAD Fixable Aqua Dead Cell staining (AmCyan; Invitrogen, 1:100). Percentage of degranulating NK92.05-CD16 cells was assessed by selecting LIVE and CD56^+^ cells, followed by gating of CD107a^+^ cells (Fig. 3A). Flow cytometry was performed on a FACSLyric, and an average of 50,000 cells was acquired for analysis.

### SARS-CoV-2 peptide pools

Overlapping (15 oligomers with 11 amino acids overlap) peptide pools spanning the entire S protein (315 peptides, PepMix; JPT) were used for the detection of WT SARS-CoV-2–specific T cell responses. To further study T cell responses to WT, B.1.1.7, and B.1.351 VOC, commercially available PepTivator Prot_S B.1.1.7 mutant pool was used. PepTivator Prot_S B.1.1.7 mutant pool covers selective mutated regions in the S protein of B.1.1.7 through 34 peptides that include deletion 69, deletion 70, deletion 144, N501Y, A570D, D614G, P681H, T716I, S982A, and D1118H. Similarly, PepTivator Prot_S B.1.351 mutant pool was used to assess cellular immune responses against B.1.351. PepTivator Prot_S B.1.351 mutant pool covers selective mutated regions in the S protein of B.1.351 through 30 peptides that include D80A, D215G, 242 deletion, 243 deletion, 244 deletion, K417N, E484K, N501Y, D614G, and A701V. For both the B.1.1.7 and B.1.351 peptide pools, a specific PepTivator WT reference pool consisting of 34 or 30 homologous peptides from the SARS-CoV-2 Wuhan strain (GenBank MN908947.3) was included as a control, respectively. All PepTivator (Miltenyi Biotec) peptide pools consist of mainly 15-nucleotide oligomers with 11 amino acids overlap.

### Ex vivo stimulations

PBMCs were thawed in R10H medium and treated with Benzonase (50 IU/ml; Merck) at 37°C for 30 min. Subsequently, 1 × 10^6^ PBMCs were stimulated with SARS-CoV-2 PepTivator or PepMix peptide pools at 1 μg/ml per peptide in 200 μl in a 96-well U-bottom plate at 37°C for 20 hours. Cells were stimulated with an equimolar concentration of DMSO (negative control) or a combination of phorbol 12-myristate 13-acetate (50 μg/ml) and ionomycin (500 μg/ml) (positive control). After stimulation, cells were stained for phenotypic lymphocyte markers. In addition, supernatants were harvested and stored at −20°C for downstream detection of cytokines.

### AIM detection

After ex vivo stimulation of PBMCs, cells were stained at 4°C for 15 min for phenotypical lymphocyte markers and AIM expression. Surface staining was performed with the following antibodies in their respective dilutions: anti–CD3-PerCP (1:25, clone SK7, BD), anti–CD4-V50 (1:50, clone L200; BD), anti–CD8–fluorescein isothiocyanate (1:25, clone DK25; Dako), anti–CD45RA-phycoerythrin (PE)–Cy7 (1:50, clone L48; BD), anti–CCR7-BV711, anti–CD69-allophycocyanin (APC)-H7 (1:50, clone FN50; BD), anti–CD137-PE (1:50, clone 4B4-1; Miltenyi), and anti–OX40-BV605 (1:25, clone L106; BD). LIVE/DEAD Fixable Aqua Dead Cell staining was included (1:100, AmCyan; Invitrogen). Cells were first gated for LIVE CD3^+^ T cells and then subdivided into CD3^+^CD4^+^ T helper cells and CD3^+^CD8^+^ T-cytotoxic cells (Fig. 4A). SARS-CoV-2–specific T cells were identified by gating the CD69^+^CD137^+^ cells (within CD4^+^ or CD8^+^ subsets). For *N* = 11 of 20 donors assessed, SARS-CoV-2 specificity in the CD4^+^ subset was confirmed by gating CD137^+^OX40^+^ cells. The DMSO-stimulated sample was used to set the cutoff gate for activation markers. On average, 500,000 cells were acquired per sample. Low-frequency samples (<10,000 cells in CD4 gate and <5000 cells in CD8 gate) were excluded from analysis.

### IFN-γ ELISA

Supernatants from PBMCs stimulated in the AIM assay were collected, and IFN-γ was measured using a commercial IFN-γ ELISA kit (QuantiFERON, Qiagen) according to the manufacturer’s instructions. Briefly, supernatants were incubated on anti–IFN-γ precoated plates in a 1:1 ratio with provided conjugate solution (1:100) at room temperature for 2 hours. An IFN-γ eight-point standard was included (0.125 to 8.000 IU/ml). Next, plates were washed and incubated with enzyme substrate solution for 20 min. The reaction was stopped by adding enzyme stopping solution and OD_450_ was measured. OD_450_ signal was corrected by subtracting background signal in the OD_620_ channel. A standard curve was generated, and IFN-γ production upon PBMC stimulation was calculated as IU/ml.

### Statistical analyses

All statistics were performed with GraphPad Prism 9.0.2. Appropriate tests were selected to compare normal or nonnormal distributed values, paired or unpaired samples, and taking into account missing values. Performed tests are indicated in the figure legends and Results.

## Supplementary Material

20210525-1

## Notes

View/request a protocol for this paper from *Bio-protocol*.
