# Supplementary material for: SARS-CoV-2 variants of concern partially escape humoral but not T cell responses in COVID-19 convalescent donors and vaccine recipients
Source: Sci Immunol. 2021 May 25;6(59):eabj1750. doi: 10.1126/sciimmunol.abj1750 (PMC9268159; doi:10.1126/sciimmunol.abj1750)
Supplement: 20210525-1 [file sciimmunol.abj1750.v1.pdf]

Cite as: D. Geers et al., Sci. Immunol. 10.1126/sciimmunol.abj1750 (2021).

CORONAVIRUS

SARS-CoV-2 variants of concern partially escape humoral but not T-cell responses in COVID-19 convalescent donors and vaccinees

Daryl Geers[1†], Marc C. Shamier[1†], Susanne Bogers[1], Gerco den Hartog[2], Lennert Gommers[1], Nella N. Nieuwkoop[1], Katharina S. Schmitz[1], Laurine C. Rijsbergen[1], Jolieke A.T. van Osch[1], Emma Dijkhuizen[1], Gaby Smits[2], Anouskha Comvalius[1], Djenolan van Mourik[1], Tom G. Caniels[3], Marit J. van Gils[3], Rogier W. Sanders[3,4], Bas B. Oude Munnink[1], Richard Molenkamp[1], Herbert J. de Jager[5], Bart L. Haagmans[1], Rik L. de Swart[1], Marion P.G. Koopmans[1], Robert S. van Binnendijk[2], Rory D. de Vries[1‡*], Corine H. GeurtsvanKessel[1‡*]

[1]Department of Viroscience, Erasmus MC; Rotterdam, the Netherlands. [2]Centre for Immunology of Infectious Diseases and Vaccines, National Institute for Public Health and the Environment; Bilthoven, the Netherlands. [3]Department of Medical Microbiology, Amsterdam UMC; Amsterdam, the Netherlands. [4]Department of Microbiology and Immunology, Weill Medical College of Cornell University; New York, NY 10021, USA. [5]Department of Occupational Health Services, Erasmus MC; Rotterdam, the Netherlands.

[†]These authors contributed equally to this work.

[‡]These authors contributed equally to this work.

***Corresponding author. Email: Rory D de Vries, r.d.devries@erasmusmc.nl; Corine H GeurtsvanKessel, c.geurtsvankessel@erasmusmc.nl**

The emergence of SARS-CoV-2 variants harboring mutations in the spike (S) protein has raised concern about potential immune escape. Here, we studied humoral and cellular immune responses to wild type SARS-CoV-2 and the B.1.1.7 and B.1.351 variants of concern in a cohort of 121 BNT162b2 mRNA-vaccinated health care workers (HCW). Twenty-three HCW recovered from mild COVID-19 disease and exhibited a recall response with high levels of SARS-CoV-2-specific functional antibodies and virus-specific T cells after a single vaccination. Specific immune responses were also detected in seronegative HCW after one vaccination, but a second dose was required to reach high levels of functional antibodies and cellular immune responses in all individuals. Vaccination-induced antibodies cross-neutralized the variants B.1.1.7 and B.1.351, but the neutralizing capacity and Fc-mediated functionality against B.1.351 was consistently 2- to 4-fold lower than to the homologous virus. In addition, peripheral blood mononuclear cells were stimulated with peptide pools spanning the mutated S regions of B.1.1.7 and B.1.351 to detect cross-reactivity of SARS-CoV-2-specific T cells with variants. Importantly, we observed no differences in $CD4^+$ T-cell activation in response to variant antigens, indicating that the B.1.1.7 and B.1.351 S proteins do not escape T-cell-mediated immunity elicited by the wild type S protein. In conclusion, this study shows that some variants can partially escape humoral immunity induced by SARS-CoV-2 infection or BNT162b2 vaccination, but S-specific $CD4^+$ T-cell activation is not affected by the mutations in the B.1.1.7 and B.1.351 variants.

INTRODUCTION

The severe acute respiratory syndrome (SARS) outbreak in 2003 was completely contained by non-pharmaceutical interventions, but controlling the spread of SARS coronavirus-2 (SARS-CoV-2) has been more difficult. Countries across the world implemented a large range of social restrictions and measures that differ in stringency and goal (*1*, *2*). A few countries have been successful in interrupting the SARS-CoV-2 transmission chain, but the majority of countries are still facing (multiple) resurgences. Implementation of long-lasting lockdowns is difficult, due to major economic and social disruption, leading to decreased compliance (*3*, *4*). A large part of the world therefore depends on the acquisition of immunity by vaccination, which, in conjunction with public health measures, should contain the coronavirus disease 2019 (COVID-19) pandemic.

It is evident that the fundamental components of the adaptive immune system (B cells, $CD4^+$ T cells and $CD8^+$ T cells) contribute to the control of SARS-CoV-2 infection (*5*–*11*). The exact correlates of protection remain to be elucidated (*12*, *13*), but circulating antibodies and memory immune cells are crucial in protection against COVID-19. Especially

important are virus-specific neutralizing antibodies targeting the receptor binding domain (RBD) of the spike (S) protein, which correlate with presence of SARS-CoV-2 specific CD4$^+$ circulating follicular helper T cells (cT_{FH}) (*8*, *14*) and can prevent the interaction between virus and the host cell (*15*). If SARS-CoV-2 establishes a reinfection, memory B and T cells rapidly proliferate and control the infection. Similarly, so-called non-neutralizing antibodies may contribute to clearance via Fc-receptor-mediated killing of virus-infected cells, a process known as antibody-dependent cellular cytotoxicity (ADCC), although this has only been shown in a limited number of studies for COVID-19 (*16*).

Immunological memory is established by an initial priming of the immune system, either by natural infection or vaccination. SARS-CoV-2 infections may induce lasting immunological memory, although the different components of the adaptive immune system exhibit distinct kinetics. Levels of S-specific IgG antibodies and virus-specific memory T cells slightly decrease over time, but levels of virus-specific memory B-cells increase over the first period of 6 months (*17*–*23*). COVID-19 vaccines were developed at an unprecedented speed, and shown to be safe and highly effective in preventing symptomatic SARS-CoV-2 infections (*24*–*27*). Exact kinetics of virus-specific immune responses induced by vaccination remain to be elucidated. Initial results indicate that S-specific binding and neutralizing antibodies slightly decline over a period of several months, although they remain detectable (*28*). Extensive characterization of the cellular immune response to vaccination and its durability is currently ongoing.

Symptomatic SARS-CoV-2 reinfections or breakthrough infections in previously infected or vaccinated subjects occur but their frequency is unknown and full evidence of reinfection is rarely provided (*29*–*31*). In the phase 3 vaccination trials almost all breakthrough infections led to mild disease, implicating that partial vaccine-induced immunity still offered protection from severe disease (*24*–*27*). However, the emergence of SARS-CoV-2 variants of concern (VOC) poses a threat: divergent strains with an accumulation of mutations in the different S domains are potentially capable of evading infection or vaccination-induced neutralizing antibodies (*32*). These VOC include the B.1.1.7 lineage that was initially detected in the United Kingdom and has now spread worldwide (*33*), but also the B.1.351 and P.1 lineages, which were detected in South Africa and Brazil respectively (*34*). These variants have a number of mutations and deletions compared to previously circulating viruses, some of which are located in the receptor binding domain (RBD). The B.1.1.7 variant acquired a substitution at amino acid 501 (N501Y), and the B.1.351 and P.1 variants additionally accumulated amino acid substitutions at positions 417 and 484 (K417N/T, E484K). Furthermore, multiple substitutions have independently evolved in the N-terminal domain (NTD) of these variants, suggesting an in vivo selective pressure on the RBD and NTD sites (*32*).

The emergence of VOC with the reduced susceptibility to polyclonal antibody responses could lead to a growing number of reinfections or breakthrough infections. In Brazil, a COVID-19 resurgence has been reported despite high seroprevalence, partially attributed to circulating strains from the P.1 and P.2 lineages (*35*, *36*). Similarly, reinfections with B.1.1.7 and B.1.351 viruses are being reported (*37*, *38*). Studies into vaccine efficacy against VOC are crucial and currently ongoing, as there is a specific concern regarding efficacy against B.1.351 and P.1. For example, the vaccination efficacy of AZD1222, which was reported to be 70% in the UK and Brazil, only reached 22% in South Africa (*39*). Reduced efficacy against B.1.351 was also reported for the NVX-CoV237 and Ad26.COV2-S vaccines by the manufacturers Novavax and Johnson & Johnson, respectively (*12*).

Although several studies have demonstrated that some VOC may be capable of evading infection or vaccination-induced neutralizing antibodies, little is known about T cell cross-reactivity with VOC. Here, we obtained serum and peripheral blood mononuclear cells (PBMC) from BNT162b2 mRNA-vaccinated health care workers (HCW) and assessed humoral and cellular immune responses to wild type (WT) SARS-CoV-2 and the B.1.1.7 and B.1.351 VOC. HCW who previously experienced COVID-19 exhibited a rapid and strong recall response upon a single vaccination, whereas seronegative HCW required two vaccinations to reach comparable levels of humoral and cellular immune responses. The B.1.351 variant was consistently less well recognized and neutralized on the antibody level, and a single vaccination in previously COVID-19 negative donors did not lead to cross-reactive neutralizing antibodies in the majority of participants. Notably, no differences in CD4$^+$ T-cell responses against WT, B.1.1.7 and B.1.351 S proteins were detected.

RESULTS

COVID-19 naive and recovered vaccination cohort

From April 2020 onwards, HCW were enrolled in a prospective cohort study upon symptomatic presentation to the occupational health services. Samples were obtained early after onset of COVID-19 symptoms (acute, T0) and 3 weeks later (convalescent, T3). Based on results from the diagnostic RT-PCR at T0 and serological screening for S-specific antibodies at T3, study participants were classified as COVID-19 naive or recovered participants. None of the participants that tested positive for COVID-19 were infected with a variant virus harboring the N501Y mutation, and none required hospitalization. From January 2021 onwards, N=121 HCW were included in a prospective vaccination study. The median age of study participants was 41 years and 9.1% were older than 60 years; 68.9% were female. The median number of days between

diagnosis (T0) and administration of the first vaccine dose was 54 days (range 23-232 days). All participants received two doses of the BNT162b2 mRNA vaccine (Pfizer/BioNTech) with an interval of 3 weeks. Among the participants, 19% (N=23) were classified as recovered from prior COVID-19. The study design is shown in **Fig. 1**, and participant characteristics are summarized in **Table 1**. Binding antibody assays were performed on samples from all 121 participants, whereas in-depth immunological analyses were performed on a selection of 25 participants (N=13 COVID-19 recovered, N=12 COVID-19 naive). The selection of participants for in-depth analysis was based on availability of longitudinal PBMC samples.

Rapid boosting of S-specific antibodies in COVID-19 recovered donors

To confirm previous SARS-CoV-2 infection, sera from the participants selected for in-depth analysis were evaluated for the presence of anti-nucleocapsid (N) immunoglobulin (Ig) antibodies during the acute and convalescent phase, and after the first (μ = 20.3 days, SD = 3.2 days) and second vaccine dose (μ = 26.5 days, SD = 5.9 days) (**Fig. 2A, Table S1**). N-specific antibodies were not detected in 11 out of 12 COVID-19 naive donors (1 low positive), whereas significant levels of N-specific antibodies were detected in 12 out of 13 COVID-19 recovered donors. As expected, N-specific antibodies were not boosted by vaccination.

Next, the presence of anti-RBD Ig and anti-S1 IgG antibodies was determined by Wantai ELISA and Luminex bead assay (MIA) (**Fig. 2B** and **2C**, **Table S1**). Absence of S-specific antibodies pre-vaccination was confirmed by both assays in the COVID-19 naive cohort, whereas S-specific antibodies were detected pre-vaccination in 22 out of 23 COVID-19 recovered donors (optical density [OD] ratio >1 in Wantai ELISA and BAU/ml > 10.08 in MIA). In some participants S-specific antibodies were already detectable at the timepoint of symptomatic testing for COVID-19 (T0). Donors selected for in-depth analysis were a good reflection of the total cohort, visualized by the color-coded symbols in **Fig. 2B** and **2C**. No significant differences were observed in binding antibody data as determined by the MIA assay between the total cohort and samples selected for in-depth analysis (**Table S2**).

After one vaccination, all COVID-19 recovered participants showed a surge in antibody levels with OD ratios >10 detected by ELISA (**Fig. 2B**, $p < 0.0001$, Friedman test). This increase was confirmed by the MIA assay (**Fig. 2C**, p = 0.0001, Friedman test, **Table S1**). The quantitatively interpretable MIA assay also showed that a second vaccination of COVID-19 recovered participants did not further boost S1-specific IgG antibodies; a plateau was reached after a single shot. In COVID-19 naive participants, 92.5% had detectable total anti-RBD Ig after one vaccine dose, but only 53.2% had an OD ratio >10 (**Fig. 2B**). In the MIA assay, all participants had detectable antibodies after 1 vaccination (**Fig. 2C, Table S1**). A clear boosting effect after the second vaccination was observed in COVID-19 naive donors. All participants then had detectable antibodies and 93.3% had a ratio >10 in ELISA, and significantly higher levels of S1-specific IgG were observed in MIA (geometric mean 252.6 ± 49.55 to 2088.5 ± 287.5 BAU/ml, $p < 0.0001$, Friedman test).

S-specific antibodies have reduced binding affinity for the B.1.351 S protein

Sera selected for in-depth analysis were initially assessed for their capacity to bind to WT (Wuhan Hu-1), B.1.1.7 and B.1.351 S proteins by ELISA at timepoints where binding antibodies were detected (post-vaccination 1 and 2, convalescent sera additionally evaluated for COVID-19 recovered participants) (**Fig. 2D**). In both COVID-19 naive and recovered donors and at all timepoints assessed, sera had reduced binding affinity for the B.1.351 S protein when compared to the WT S protein. Additionally, at two timepoints a slightly increased binding affinity for B.1.1.7 S was observed ($Vx^1 3$ for naive donors and T3 for convalescent donors).

Neutralizing antibodies have reduced activity against B.1.351

Sera selected for in-depth analysis were subsequently tested for the presence of neutralizing antibodies by an infectious virus plaque reduction neutralization test ($PRNT_{50}$) at timepoints when binding antibodies were detected by ELISA (post-vaccination 1 and 2, convalescent phase additionally evaluated for COVID-19 recovered participants) (**Fig. 2E**, individual S-curves in **Fig. S1**). In COVID-19 naive HCW, a single vaccine dose led to detectable levels of neutralizing antibodies in 7 out of 12 donors. $PRNT_{50}$ values were boosted by a second vaccination to detectable levels in all donors (p = 0.0005, Wilcoxon test, **Table S1**), but the peak titer was significantly lower compared to COVID-19 recovered participants (geometric mean titer 1:189, $p < 0.0001$, unpaired t test). Neutralizing antibodies against WT SARS-CoV-2 (which contained the D614G mutation) were detected in all sera collected from N=13 COVID-19 recovered HCW prior to vaccination. A single vaccination boosted the $PRNT_{50}$ titers to a geometric mean plateau value of 1:1874 ($p < 0.0001$, RM one-way ANOVA). Titers did not increase further after a second vaccination.

Next, cross-reactivity of neutralizing antibodies induced by vaccination or infection against VOC was evaluated (**Fig. 2F**). In pre-vaccination sera from COVID-19 recovered donors, neutralizing antibodies against WT SARS-CoV-2 (D614G), B.1.1.7 and B.1.351 were detected in 13 out of 13, 12 out of 13, and 7 out of 13 donors, respectively (**Table S1**). A single vaccination was sufficient to boost neutralizing antibodies to detectable levels for all SARS-CoV-2 variants, and a second dose did not further boost antibody titers. Compared to $PRNT_{50}$ titers against WT SARS-CoV-2, geometric mean titers against B.1.1.7 were consistently higher (2.5-fold and 2.2-

fold increase post-vaccination 1 and 2, $p = 0.0114$ and $p < 0.0001$ [RM one-way ANOVA], respectively), whereas titers against B.1.351 were consistently lower (2.7-fold and 3.3-fold decrease post-vaccination 1 and 2, $p = 0.0004$ and $p < 0.0001$ [RM one-way ANOVA], respectively) (**summarized in Table S3**). Neutralizing antibodies were detected after 1 vaccination in 7 out of 12, 6 out of 12, and 2 out of 12 COVID-19 naive donors against WT SARS-CoV-2, B.1.1.7 and B.1.351, respectively. A second vaccine dose boosted that to 12 out of 12, 11 out of 12, and 10 out of 12. Geometric mean titers after the first vaccination against all VOC were not significantly different, but after the second vaccination a 2-fold increase of neutralizing antibodies against B.1.1.7 and a 3.1-fold decrease against B.1.351 was detected ($p = 0.0013$ and $p < 0.0001$, respectively, RM one-way ANOVA) (**summarized in Table S3**).

Antibodies have reduced Fc-mediated functionality against B.1.351

Next, sera were evaluated for the presence of antibodies that could activate NK cells as a proxy for ADCC. A set dilution of serum (1:100) was incubated on plates coated with His-tagged proteins (WT N, WT S [Wuhan Hu-1], B.1.1.7 S and B.1.351 S), followed by addition of an immortalized FcγRIII$^+$ NK cell line. Activation of NK cells was measured by flow cytometry by detecting lysosomal-associated membrane protein-1 (LAMP-1 or CD107a$^+$, gating strategy shown in **Fig. 3A**). N-specific ADCC-mediating antibodies were not detected in the N=12 COVID-19 naive donors at any timepoint (**Fig. 3B, Table S1**). In N=13 COVID-19 recovered donors, N-specific ADCC-mediating antibodies were detected in the convalescent phase initially. These N-specific antibodies gradually waned over time (convalescent versus post-vaccination 2: $p = 0.005$, Friedman test). As expected, vaccination did not boost N-specific antibodies.

In COVID-19 naive donors, WT S-specific ADCC-mediating antibodies were not detected pre-vaccination, whereas in COVID-19 recovered donors WT S-specific ADCC-mediating antibodies were detected in the convalescent phase (**Fig. 3C**, $p = 0.0038$, RM one-way ANOVA, **Table S1**). In COVID-19 naive donors, the first vaccination led to low-level detection of ADCC-mediating antibodies, which were further boosted by the second vaccination. In COVID-19 recovered donors, ADCC-mediating antibodies were already boosted by a single vaccination to peak levels and the second shot did not lead to an additional boosting (reminiscent of binding and neutralizing antibodies, see **Fig. 2**).

Percentages of degranulating NK cells were comparable between WT and B.1.1.7 S at all timepoints, regardless of whether donors had been previously exposed to SARS-CoV-2 or not, and the number of vaccinations. However, ADCC-mediating antibodies had significantly reduced activity against B.1.351 S at almost all timepoints in COVID-19 naive and recovered donors (**Fig. 3D**, tested by mixed-effect models). Notably, even in recovered donors after two vaccinations this reduced activity to B.1.351 was apparent. Percentages of CD107a$^+$ NK cells were significantly correlated to the binding antibody titers (shown in **Fig. 2D**), however the correlation was not evident at all timepoints assessed separately (**Fig. S2**).

Rapid boosting of S-specific T cells in COVID-19 recovered donors

Besides serological responses, we assessed the presence of S-specific T-cell responses in COVID-19 naive (N=7) and recovered (N=13) HCW in the acute and convalescent phase, and after vaccination. To this end, PBMC were stimulated with either overlapping peptide pools representing the full-length WT S protein (Wuhan Hu-1) or peptide pools covering the selected mutated regions in the S protein from the B.1.1.7 and B.1.351 VOC. Following stimulation, activation-induced marker (AIM, CD69 and CD137) expression within CD4$^+$ and CD8$^+$ subsets was measured by flow cytometry (gating strategy shown in **Fig. 4A**). Up-regulation of OX40 and CD137 was additionally assessed in the CD4$^+$ subset from N=11 donors and correlated significantly to the up-regulation of CD69 and CD137 (**Fig. S3**).

In COVID-19 naive donors, WT S-specific CD4$^+$ T cells expressing AIM (CD69$^+$CD137$^+$) were detected after the first and second vaccination (**Fig. 4B,** T3 to Vx^{1}3: $p = 0.0374$, T3 to Vx^{2}3: $p = 0.0016$, Kruskal-Wallis test). In COVID-19 recovered donors, S-specific CD4$^+$ T cells were already detected in the convalescent phase, which were boosted after the first vaccination. A second vaccination did not lead to an additional boosting effect (**Fig. 4B**). Similar results were observed when a stimulation index was calculated (SI, **Fig. 4C**, ratio of S-specific CD4$^+$ T-cell activation over background activation). Previously, we regarded individuals with a SI > 3 as responders, however this was in an intensive care unit (ICU) cohort with strong T-cell responses (*11*). Since the strength of the T-cell response seems to be correlated to disease severity (*40*, *41*), we lowered the arbitrary cut-off to an SI > 2 to identify responders after mild COVID-19 (**Table S1**). In COVID-19 naive donors, CD4$^+$ T-cell responders were not observed pre-vaccination, whereas 5 out of 7 responders were identified after 1 or 2 vaccinations. In COVID-19 recovered donors, 8 out of 12 responders were identified pre-vaccination, which increased to 10 out of 12 responders after 1 or 2 vaccinations.

CD8$^+$ S-specific T-cell responses in COVID-19 naive (N=7) and recovered (N=13) HCW were more difficult to detect. In COVID-19 naive participants pre-vaccination, CD8$^+$ T-cell responses were never observed. A non-significant trend for increasing CD8$^+$ T-cell responses (both AIM and SI, **Fig. 4D**, data at T0 from COVID-19 recovered donors lacking due to low counts in CD8$^+$ gate) was observed post-vaccination of COVID-19 negative donors. In COVID-19 recovered participants, CD8$^+$ S-specific T cells were already observed in the

convalescent phase, with increased levels after two vaccinations (p = 0.046, Wilcoxon rank test).

S-specific CD4⁺ T cells have comparable reactivity with VOC

Subsequently, we assessed T-cell responses to VOC B.1.1.7 and B.1.351. Instead of using overlapping peptide pools covering the full S protein, we used commercially available peptide pools that specifically covered the mutated regions, and compared the responses with the corresponding WT control pools containing homologous peptides derived from the WT strain. With these pools, S-specific $CD4^+$ T-cell responses were again detected (SI > 2) in the post-vaccination samples from COVID-19 naive samples, and in the convalescent and post-vaccination samples of COVID-19 recovered donors (**Fig. 4E** and **4F**, shown as percentages of activated cells within the $CD4^+$ T-cell subset in **Fig. S4**), but T-cell responses were not observed prior to vaccination in the COVID-19 naive participants. Notably, in both COVID-19 naive and recovered participants, no differences were observed between $CD4^+$ T-cell responses to WT S and B.1.1.7 S (**Fig. 4E**, **Fig. S4A**), or between WT S and B.1.351 S (**Fig. 4F**, **Fig. S4B**). Additionally, we measured the production of IFNγ in cell culture supernatant in response to WT and mutant pools in a subset of samples and did not observe any significant differences in $CD4^+$ T-cell reactivity to mutant S peptide pools (**Fig. S5**). Since we observed minimal SARS-CoV-2-specific $CD8^+$ T-cell responses, we did not draw any conclusions on $CD8^+$ T-cell responses cross-reactive to VOC.

DISCUSSION

Here, we show that both in COVID-19 recovered and naive individuals BNT162b2 mRNA vaccination induces robust SARS-CoV-2-specific neutralizing antibodies, ADCC-mediating antibodies and $CD4^+$ and $CD8^+$ T cells. In line with previous studies (*42*), a single vaccination led to a rapid and strong recall responses in COVID-19 recovered participants, without detectable boosting after the second dose. In COVID-19 naive individuals, a second dose was required to consistently detect neutralizing antibodies. Vaccination-induced antibodies did cross-neutralize the variants B.1.1.7 and B.1.351, but the neutralizing capacity and Fc-mediated functionality against B.1.351 was consistently 2- to 4-fold lower than to the homologous virus. Finally, we detected SARS-CoV-2-specific $CD4^+$ and $CD8^+$ T cells after infection and vaccination, and $CD4^+$ T cell activation was robust and appeared indifferent to the S mutations of the B.1.1.7 and B.1.351 variants.

The study was performed in a selected population of HCW working with COVID-19 patients who were eligible for rapid vaccination in the Netherlands. The participants were relatively young, predominantly female, with a history of mild COVID-19 disease if previously infected. The population was underpowered to compare vaccination responses in distinct subsets of participants (*e.g.*, age groups, comorbidity). Future studies are required to analyze responses in these specific populations, and more specifically in vulnerable populations at risk for a less efficient vaccination response (*43*).

Serological screening assays ideally correlate with the neutralizing capacity of detected SARS-CoV-2-specific antibodies. Although the $PRNT_{50}$ with infectious virus is considered the gold standard to measure neutralizing antibody titers, its laborious nature at high biosafety containment level makes it an inappropriate tool for testing of large sample sets and surveillance purposes. To detect antibodies in the complete HCW cohort we have therefore used well-validated high-throughput assays targeting the S1 or RBD regions of the S protein (Wantai total Ig ELISA and Luminex microsphere immunoassay or MIA). Although the Wantai ELISA is a qualitative assay, a ratio of > 10 has previously been shown to correspond to virus neutralization (*44*). Since this sensitive assay is often used in diagnostic laboratories, we show here that the presence of antibodies in response to vaccination (in the absence or presence of pre-existing SARS-CoV-2 immunity) can indeed be measured, but not accurately quantified. The Luminex MIA on the other hand allowed clear interpretation and quantitative comparison of antibody responses within the groups and at different timepoints (*45*).

Although seroconversion was observed in all groups after vaccination, the COVID-19 naive group required a second vaccination to obtain neutralizing titers equal to or higher than the cut-off titer of 1:20. Even after the second vaccination, COVID-19 naive participants had lower neutralizing antibody titers compared to COVID-19 recovered participants (after 1 vaccination). In hospitalized patients, we have previously shown that a serum neutralizing antibody titer of at least 1:20 was independently associated with absence of shedding of infectious SARS-CoV-2 (*46*). The neutralization data reported in this study is in line with previous reports (*42*, *47*–*49*).

The B.1.1.7 and B.1.351 variants both contain the signature mutation N501Y, associated with increased affinity for the ACE2 receptor. We ascertained that none of the COVID-19 recovered subjects in our study had been infected by SARS-CoV-2 harboring this N501Y mutation and that therefore the observed neutralization against VOC in this study was based on cross-reactivity and not on previous priming with a B.1.1.7 or B.1.351 virus. Concerning neutralization of VOC, recent studies have consistently reported lower neutralizing antibody titers against B.1.351 after vaccination, but not against B.1.1.7 (*39*, *42*, *48*–*59*). In our study, we detected consistently increased neutralizing antibody titers in polyclonal sera against B.1.1.7 when compared to WT, both after SARS-CoV-2 infection and vaccination. In some sera we observed increased binding to B.1.1.7 S by ELISA as well, however not at all timepoints therefore not explaining the increased neutralization. Our study differs from most previously conducted

studies because infectious viruses were used to determine neutralizing antibody titers (in contrast to pseudotyped viruses), potentially explaining this observation. We also observed lower serum neutralization efficiency of the B.1.351 VOC, but none of the sera of vaccinated subjects in our cohort showed a complete escape of this variant. Future studies are required to evaluate to what extent the differences in neutralizing antibody titers correlate with the risk of breakthrough infections.

In addition to direct virus neutralization, antibodies can have multiple other modes of action that are primarily mediated by immunoglobulin (Ig) IgG1 and IgG3 subclass antibodies (*60*). After binding to antigens displayed on virus-infected cells, the Fc domain of the antibody engages Fc receptors on effector cells that subsequently kill virus-infected cells, a process known as ADCC. ADCC is mainly mediated by the interaction between virus-specific antibodies and Fcγ receptor IIIα (FcγRIIIα, CD16), which is for example present on natural killer (NK) cells (*61*–*63*). Little is known about the role of ADCC-mediating antibodies in COVID-19, but NK cells were shown to be decreased in COVID-19 patients in association with disease severity (*64*). In ex vivo studies, NK cells from COVID-19 patients displayed impaired cytotoxicity (*64*, *65*), potentially due to cytokine dysregulation and high plasma levels of IL-6 and TNF-α (*66*). ADCC-mediating antibodies have been detected in plasma of COVID-19 convalescent donors (*16*, *67*, *68*). We show that S-specific ADCC-mediating antibodies are induced both by infection and vaccination, but we detect reduced NK cell activation in response to binding to the B.1.351 S protein. Although this reduced activation directly correlated to reduced binding of sera to VOC antigens, a significant correlation was often not observed in sera collected upon multiple exposures to the S antigen. Therefore, the quantity of S-binding antibodies does not fully explain the reduced Fc-mediated effector functionality. In addition to measuring NK cell activation by specific antibodies, future studies should measure functional cytotoxicity using the combination of infectious virus and primary cells.

Whereas previous studies have focused on potential immune evasion by VOC on the antibody level, little is known about immune escape at the T-cell level. A small study assessed 45 mutations in the B.1.351 S protein, and found that only 1 mutation overlapped with a low-prevalent $CD8^+$ T-cell epitope (*69*). A study that followed a sequencing-based approach identified mutations in $CD8^+$ T-cell epitopes after sequencing 747 SARS-CoV-2 isolates, and showed that tetramer-sorted $CD8^+$ T cell clones responded to mutant peptides in a transcriptionally different manner (*70*). We performed a comprehensive flow cytometry analysis of SARS-CoV-2-specific $CD4^+$ and $CD8^+$ T cells from COVID-19 naive and recovered donors, before and after mRNA vaccination. In our analyses, S-specific $CD4^+$ and $CD8^+$ T cells were induced or boosted by the BTN162b2 vaccine, and the induced $CD4^+$ T cells equally recognized the WT, B.1.1.7 and B.1.351 S proteins. This is in line with one other recently performed study, that additionally showed cross-reactivity to the P.1 and CAL.20C (B.1.429) variants (*71*). Our study on specific T cells has some limitations: (1) the sample size for which in-depth T-cell profiling was performed was limited to 20 donors; (2) we only focused on S-specific T cells; and (3) we were not able to further address $CD8^+$ T-cell responses to VOC, due to low frequency of SARS-CoV-2-specific $CD8^+$ T cells. Although we hypothesize this was due to minimal disease severity in this HCW cohort (*40*, *41*), alternatively this could be because of limited sensitivity when using peptide pools containing overlapping 15mers. Additional studies with smaller peptides (8-10mers) predicted or shown to bind HLA class I are advised to specifically study VOC cross-reactive $CD8^+$ T-cell responses. In some donors, we also observed the presence of SARS-CoV-2 S-specific T cells prior to exposure to antigen. This is probably due to the detection of seasonal coronavirus (HCoV)-specific T cells, cross-reactive with SARS-CoV-2. Detection of cross-reactive T cells via the AIM assay was previously described (*7*, *11*, *72*). Although verification of our methods with a potentially more discriminative assay like IFNγ ELISPOT (*73*–*75*) could be useful, the sensitive AIM assay may be the best suited assay to detect small differences in reactivity to different S antigens.

We generated extensive immunological response profiles against WT SARS-CoV-2, and VOC B.1.1.7 and B.1.351. However, additional VOC and variants of interest (VOI) are continuously emerging (*76*). We did not analyze immune responses to the P.1 VOC, originating from Brazil, which also contains mutations in the RBD at positions 417 and 484 (in combination with unique mutations throughout the S protein). Although this variant was not investigated in the current study, previous studies have demonstrated a similar, less pronounced, decrease in neutralization by polyclonal sera after BNT162b2 vaccination (*47*, *77*). Little is known about cross-reactivity on the T-cell level to this VOC. Future studies should also focus on complete and in-depth immunological profiling of VOC and VOI.

In conclusion, this study emphasizes the importance of a complete assessment of functional immune responses to VOC or variants or interest. We confirm that a single mRNA BNT162b2 vaccination is sufficient to induce vigorous immune responses in previously COVID-19 recovered individuals, on both the humoral and cellular level. Additionally, we show that polyclonal sera have reduced functionality against VOC B.1.351, but that $CD4^+$ T-cell activation in response to the S protein of this variant (and VOC B.1.1.7) was robust. On basis of these results, we hypothesize that the reduced antibody responses to VOC lead to an increased risk of breakthrough infections with these variants, especially when antibody titers

wane after vaccination. However, protection against severe disease caused by these VOC may still be provided by cross-reactive SARS-CoV-2-specific T-cell-mediated immunity. Future studies are required to assess to what extent sterile immunity is a requirement to reach herd immunity and interrupt SARS-CoV-2 circulation. Continuous surveillance will monitor the incidence of breakthrough infections and duration of vaccine-induced immunity.

MATERIALS AND METHODS

Study design

The Erasmus MC COVID-19 health care workers study was approved by the institutional review board (medical ethical committee, MEC-2020-0264). Informed consent was obtained from all participants. At an early stage in the COVID-19 pandemic, N=121 symptomatic HCW presenting to the occupational health services from Erasmus MC, Rotterdam, the Netherlands, were enrolled into a prospective cohort study. Serum samples were obtained from all participants during the acute phase (T0, time of diagnostic RT-PCR) and the convalescent phase (T3, 3 weeks after RT-PCR); additional PBMC were obtained from N=20 participants. Based on the diagnostic RT-PCR result at T0 and serology result at T3, study participants were classified as COVID-19 naive (N=98) or recovered (n=23) participants. As soon as vaccines became available for selected groups of HCW, starting in January 2021, study participants were enrolled into a vaccination study. Participants received two doses of the BNT162b2 (Pfizer/BioNTech) mRNA vaccine with an interval of 21 days. Follow up samples were collected 14-21 days after the first dose and 21-28 days after the second dose. Study design is also shown in **Fig. 1**.

RT-PCR assays for the detection of SNPs associated with SARS-CoV-2 VOC

The deletion in spike protein (HV69-70) that is indicative of the B.1.1.7 variant was detected by the Applied Biosystems TaqPath COVID-19 kit (ThermoFisher Scientific). The assay was performed according to the instructions of the manufacturer. This COVID-19 detection kit combines RT-PCR on the Orf1ab, N and S genes. Failure of detection of the S-gene in combination with detection of SARS-CoV-2 RNA for the Orf1ab and N-genes is indicative for the HV69-70 deletion in spike (S gene Target Failure, SGTF) (*78*). In addition, the presence of the asparagine to tyrosine mutation at spike protein position 501 (N501Y) was detected by the VirSNiP N501Y assay (TIB Molbiol A23063T) according to the instructions of the manufacturer. The combination of delHV69-70 and N501Y was considered to be indicative for B1.1.7. Detection of N501Y alone was considered to be indicative for B.1.351.

PBMC and plasma isolation

Serum was collected in 10 ml tubes without anti-coagulant, centrifuged at 2500 rpm for 15 min, aliquoted and stored at -20°C for further experiments. Peripheral blood mononuclear cells (PBMC) were isolated from blood collected in K_3EDTA tubes by density gradient centrifugation. Briefly, blood collection tubes were centrifugated at 2500 rpm for 15 min after which plasma was aliquoted and stored at -20°C. The cellular fraction was diluted in phosphate buffered saline (PBS), layered on a density gradient (Lymphoprep, Stemcell Technologies) and PBMC were separated by centrifugating at 2000 rpm for 30 min. PBMC were washed 4 times in PBS and frozen in 90% fetal bovine serum (FBS) with 10% DMSO (Honeywell) at -135°C until use in stimulation assays.

RBD/Wantai and N-specific antibody ELISA

Humoral immune responses to vaccination were analyzed using a qualitative ELISA for the detection of total antibodies against the SARS-CoV-2 receptor binding domain (RBD) (Beijing Wantai Biological Pharmacy Enterprise Co., Ltd.) as described previously (*44*). Optical Density (OD) ratios above 1.0 were interpreted as positive as indicated by the manufacturer. In this assay, ratios above 10 are indicative of the presence of neutralizing antibodies.

Antibody levels against nucleocapsid (N) and spike (S) in serum were measured by performing an in-house developed ELISA. ELISA plates were coated with a His-tagged N or S protein (25 or 20 ng/well, respectively) at 4°C overnight. Following coating, plates were blocked, washed and incubated with a dilution series of plasma (1:40 to 1:2560 for N, data from 1:40 used in **Fig. 2A;** 1:20 to 1:163840 for S) at 37°C for 2 hours, after which plates were washed and horseradish peroxidase (HRP)–labeled rabbit anti-human IgG (1:6000, Dako) was added. Plates were incubated at 37°C for 1 hour, washed and developed by using 3,3′,5,5′-tetramethylbenzidine (KPL). Plates were measured at an optical density of 450 nm (OD450) using an ELISA microtiter plate reader. OD450 signal was corrected by subtracting background signal in the OD620 channel. The cut-off was set at 3x average background of multiple negative control sera. For the S ELISA an arbitrary cut-off value was set at OD450 0.300. which was used to calculate an endpoint titer.

Luminex bead assay

Serum samples were tested for the presence of IgG antibodies to SARS-CoV-2 S1 using a previously published fluorescent bead-based immune assay (*45*). The specificity (99.7%) and sensitivity (91.6%) of the assay was determined using a heterogeneous sample set including asymptomatic, and mild to severe COVID-19 cases as representative of COVID-19 cases in the general population, as well pre-pandemic population samples and samples of persons infected with various viruses including endemic coronaviruses, as negative controls (*23*). Concentrations were interpolated from a reference consisting of pooled sera using a 5-parameter logistic fit and NIBSC/WHO COVID-19 reference serum 20/136, and expressed as international Binding Antibody

Units per ml (BAU/ml). A BAU/ml value of >10,08 was considered positive.

Plaque reduction neutralization assay

We used the plaque reduction neutralization test (PRNT) in which we tested serum samples for their neutralization capacity against SARS-CoV-2 variants as previously described (*9*). Viruses used in the assay were isolated from diagnostic specimen at the department of Viroscience, Erasmus MC, cultured and subsequently sequenced to rule out additional mutations in the S protein: D614G (GISAID: hCov-19/Netherlands/ZH-EMC-2498), B.1.1.7 (GISAID: hCov-19/Netherlands/ZH-EMC-1148) and B.1.351 (GISAID: hCov-19/Netherlands/ZH-EMC-1461). Heat-inactivated sera were 2-fold diluted in Dulbecco modified Eagle medium supplemented with $NaHCO_3$, HEPES buffer, penicillin, streptomycin, and 1% fetal bovine serum, starting at a dilution of 1:10 in 60 μL. We then added 60 μL of virus suspension to each well and incubated at 37°C for 1 hour (leading to ±1,000 plaques/well in infection controls). After 1 hour incubation, we transferred the mixtures on to Vero-E6 cells and incubated for 8 hours. After incubation, we fixed the cells with 10% formaldehyde and stained the cells with polyclonal rabbit anti-SARS-CoV-2 nucleocapsid antibody (Sino Biological) and a secondary peroxidase-labeled goat anti-rabbit IgG (Dako). We developed signal by using a precipitate-forming 3,3′,5,5′-tetramethylbenzidine substrate (TrueBlue; Kirkegaard & Perry Laboratories) and counted the number of infected cells per well by using an ImmunoSpot Image Analyzer (CTL Europe GmbH). The dilution that would yield 50% reduction of plaques ($PRNT_{50}$) compared to the infection control (included on all plates) was estimated by determining the proportionate distance between two dilutions, from which an endpoint titer was calculated. We considered a titer >20 to be positive based on assay validation.

PBMC and NK92.05 cell culture

Experiments with PBMC were performed in Gibco Roswell Park Memorial Institute (RPMI) 1640 Medium (Gibco) supplemented with 10% human serum (Sanquin, Rotterdam), penicillin (100 IU/mL; Lonza, Belgium), streptomycin (100 μg/mL; Lonza, Belgium), and 2 mM L-glutamine (Lonza, Belgium; R10H medium). Antibody-dependent cell-mediated cytotoxicity experiments were performed with the FcγRIII (CD16)-expressing NK92.05 natural killer (NK) cell-line (NK92.05-CD16). The NK92.05-CD16 continuous cell-line was a kind gift from Kerry S. Campbell at the Fox Chase Cancer Center in Pennsylvania (*79*). NK92.05-CD16 cells were cultured in sterile filtered alpha-MEM supplemented with sodium bicarbonate (2.2 g/L, pH 7.2), 2-ME (0.0001 M), L-glutamine (200 mM, Gibco), myo-inositol (0.2 mM), 10% horse serum, 10% fetal bovine serum, folic acid (0.004 mM), sodium pyruvate (1 mM), penicillin (100 IU/mL), and streptomycin (100 μg/mL). Cells were cultured in the presence of human recombinant IL-2 (100 IU/mL). CD16 expression in this cell-line was monitored by flow cytometry by staining with anti-CD16-AF647 (clone 3G8, Southern Biotech, 1:50).

ADCC assay

The presence of ADCC-mediating antibodies in plasma was measured using soluble pre-fusion stabilized His-tagged S proteins. The constructs contained the following mutations compared to the WT variant (Wuhan Hu-1; GenBank: MN908947.3): deletion (Δ) of H69, V70 and Y144, N501Y, A570D, D614G, P681H, T716I, S982A and D1118H in B.1.1.7; L18F, D80A, D215G, L242H, K417N, E484K, N501Y, D614G and A701V in B.1.351. All S constructs were cloned into a pPPI4 expression vector containing a hexahistidine (His) tag, verified by Sanger sequencing and subsequently produced in HEK293F cells and purified as previously described (*6*, *80*). ADCC was also assessed against SARS-CoV-2 His-tagged full-length N (Sino Biological). ADCC assay was performed as previously described (*61*). In short, high-binding 96-well flat bottom plates (Immunolon) were coated with 200 ng SARS-CoV-2 S or N per well, incubated at 4°C overnight, blocked in 5% BSA, washed with PBS and incubated with plasma (1:100) at 37°C for 2 hours. Plates were washed in PBS and 100,000 NK92.05-CD16 cells were added in combination with anti-CD107a-V450 (clone H4A3, BD, 1:100), GolgiStop (0.67 μL/mL, BD) and golgiplug (1 μL/mL, BD). Plates were incubated at 37°C for 5 hours. Cells were subsequently stained with anti-CD56-PE (clone B159, BD, 1:25) and LIVE/DEAD Fixable Aqua Dead Cell staining (AmCyan, Invitrogen, 1:100). Percentage of degranulating NK92.05-CD16 cells was assessed by selecting LIVE and $CD56^+$ cells, followed by gating of $CD107a^+$ cells (**Fig. 3A**). Flow cytometry was performed on a FACSLyric and an average of 50,000 cells was acquired for analysis.

SARS-CoV-2 peptide pools

Overlapping (15-mers with 11 amino acids overlap) peptide pools spanning the entire S-protein (315 peptides, JPT, PepMix) were used for the detection of WT SARS-CoV-2-specific T-cell responses. To further study T-cell responses to WT, B.1.1.7 and B.1.351 VOC, commercially available PepTivator® Prot_S B.1.1.7 mutant pool was used. PepTivator® Prot_S B.1.1.7 mutant pool covers selective mutated regions in the S protein of B.1.1.7 through 34 peptides that include deletion 69, deletion 70, deletion 144, N501Y, A570D, D614G, P681H, T716I, S982A, and D1118H. Similarly, PepTivator® Prot_S B.1.351 mutant pool was used to assess cellular immune responses against B.1.351. PepTivator® Prot_S B.1.351 mutant pool covers selective mutated regions in the S protein of B.1.351 through 30 peptides that include D80A, D215G, 242 deletion, 243 deletion, 244 deletion, K417N, E484K, N501Y, D614G, and A701V. For both the B.1.1.7 and B.1.351 peptide pools a specific PepTivator® WT reference pool consisting of 34 or 30 homologous peptides from the SARS-CoV-2 Wuhan

strain (GenBank MN908947.3) was included as a control, respectively. All PepTivator® (Miltenyi) peptide pools consist of mainly 15-mers with 11 amino acids overlap.

Ex vivo stimulations

PBMC were thawed in R10H medium and treated with Benzonase® (50 IU/mL; Merck) at 37°C for 30 min. Subsequently, 1×10^6 PBMC were stimulated with SARS-CoV-2 PepTivator® or PepMix peptide pools at 1μg/mL per peptide in 200μl in a 96-well U-bottom plate at 37°C for 20 hours. Cells were stimulated with an equimolar concentration of DMSO (negative control) or a combination of PMA (50 μg/mL) and ionomycin (500 μg/mL) (positive control). Following stimulation, cells were stained for phenotypic lymphocyte markers. Additionally, supernatants were harvested and stored at -20°C for downstream detection of cytokines.

Activation-induced marker (AIM) detection

Following ex vivo stimulation of PBMC, cells were stained at 4°C for 15 min for phenotypical lymphocyte markers and AIM expression. Surface staining was performed with the following antibodies in their respective dilutions: anti-CD3-PerCP (Clone SK7, BD, 1:25), anti-CD4-V50 (Clone L200, BD, 1:50), anti-CD8-FITC (Clone DK25, Dako, 1:25), anti-CD45RA-PE-Cy7 (Clone L48, BD, 1:50), anti-CCR7-BV711, anti-CD69-APC-H7 (Clone FN50, BD, 1:50), anti-CD137-PE (Clone 4B4-1, Miltenyi, 1:50), and anti-OX40-BV605 (Clone L106, BD, 1:25). LIVE/DEAD Fixable Aqua Dead Cell staining was included (AmCyan, Invitrogen, 1:100). Cells were first gated for LIVE $CD3^+$ T cells and then subdivided into $CD3^+CD4^+$ T-helper cells and $CD3^+CD8^+$ T-cytotoxic cells (**Fig. 4A**). SARS-CoV-2-specific T cells were identified by gating the $CD69^+CD137^+$ cells (within $CD4^+$ or $CD8^+$ subsets). For N=11 out of 20 donors assessed, SARS-CoV-2 specificity in the $CD4^+$ subset was confirmed by gating $CD137^+OX40^+$ cells. The DMSO-stimulated sample was used to set the cutoff gate for activation markers. On average 500,000 cells were acquired per sample. Low frequency samples (<10,000 cells in CD4 gate, <5,000 cells in CD8 gate) were excluded from analysis.

Interferon-gamma (IFNγ) ELISA

Supernatants from PBMC stimulated in the AIM assay were collected and IFNγ was measured using a commercial IFNγ ELISA kit (QuantiFERON, Qiagen) according to the manufacturer's instructions. Briefly, supernatants were incubated on anti-IFNγ precoated plates in a 1 to 1 ratio with provided conjugate solution (1:100) at room temperature for 2 hours. An IFNγ 8-point standard was included (0.125 IU/mL to 8.000 IU/mL). Next, plates were washed and incubated with enzyme substrate solution for 20 min. The reaction was stopped by adding enzyme stopping solution and OD450 was measured. OD450 signal was corrected by subtracting background signal in the OD620 channel. A standard curve was generated and IFNγ production upon PBMC stimulation was calculated as IU/mL.

Statistical analyses

All statistics were performed with GraphPad Prism 9.0.2. Appropriate tests were selected to compare normal or non-normal distributed values, paired or unpaired samples, and taking into account missing values. Performed tests are indicated in the figure legends and results sections.

SUPPLEMENTARY MATERIALS

immunology.sciencemag.org/cgi/content/full/6/59/eabj1750/DC1

Figure S1. Normalized S-curves for determining $PRNT_{50}$
Figure S2. Correlation between antibody binding and Fc-mediated functionality
Figure S3. Detection of SARS-CoV-2-specific T cells using OX40
Figure S4. Detection of S-specific T cells by measuring upregulation of activation-induced markers (AIM)
Figure S5. Detection of S-specific T cells by measuring production of IFNγ in culture supernatant
Table S1. Overview of immunological responders
Table S2: Comparison of geometric mean S1-specific IgG BAU/ml (MIA) between in-depth analyzed sera and all sera
Table S3. $PRNT_{50}$ fold change reactivity to VOC
Data file S1. Raw data file (Excel spreadsheet)

REFERENCES AND NOTES

1. T. Hale, N. Angrist, R. Goldszmidt, B. Kira, A. Petherick, T. Phillips, S. Webster, E. Cameron-Blake, L. Hallas, S. Majumdar, H. Tatlow, A global panel database of pandemic policies (Oxford COVID-19 Government Response Tracker). *Nat. Hum. Behav.* 5, 529–538 (2021). doi:10.1038/s41562-021-01079-8 Medline
2. A. Robert, Lessons from New Zealand's COVID-19 outbreak response. *Lancet Public Health* 5, e569–e570 (2020). doi:10.1016/S2468-2667(20)30237-1 Medline
3. C. Maringe, J. Spicer, M. Morris, A. Purushotham, E. Nolte, R. Sullivan, B. Rachet, A. Aggarwal, The impact of the COVID-19 pandemic on cancer deaths due to delays in diagnosis in England, UK: A national, population-based, modelling study. *Lancet Oncol.* 21, 1023–1034 (2020). doi:10.1016/S1470-2045(20)30388-0 Medline
4. M. Pierce, H. Hope, T. Ford, S. Hatch, M. Hotopf, A. John, E. Kontopantelis, R. Webb, S. Wessely, S. McManus, K. M. Abel, Mental health before and during the COVID-19 pandemic: A longitudinal probability sample survey of the UK population. *Lancet Psychiatry* 7, 883–892 (2020). doi:10.1016/S2215-0366(20)30308-4 Medline
5. J. Braun, L. Loyal, M. Frentsch, D. Wendisch, P. Georg, F. Kurth, S. Hippenstiel, M. Dingeldey, B. Kruse, F. Fauchere, E. Baysal, M. Mangold, L. Henze, R. Lauster, M. A. Mall, K. Beyer, J. Röhmel, S. Voigt, J. Schmitz, S. Miltenyi, I. Demuth, M. A. Müller, A. Hocke, M. Witzenrath, N. Suttorp, F. Kern, U. Reimer, H. Wenschuh, C. Drosten, V. M. Corman, C. Giesecke-Thiel, L. E. Sander, A. Thiel, SARS-CoV-2-reactive T cells in healthy donors and patients with COVID-19. *Nature* 587, 270–274 (2020). doi:10.1038/s41586-020-2598-9 Medline
6. P. J. M. Brouwer, T. G. Caniels, K. van der Straten, J. L. Snitselaar, Y. Aldon, S. Bangaru, J. L. Torres, N. M. A. Okba, M. Claireaux, G. Kerster, A. E. H. Bentlage, M. M. van Haaren, D. Guerra, J. A. Burger, E. E. Schermer, K. D. Verheul, N. van der Velde, A. van der Kooi, J. van Schooten, M. J. van Breemen, T. P. L. Bijl, K. Sliepen, A. Aartse, R. Derking, I. Bontjer, N. A. Kootstra, W. J. Wiersinga, G. Vidarsson, B. L. Haagmans, A. B. Ward, G. J. de Bree, R. W. Sanders, M. J. van Gils, Potent neutralizing antibodies from COVID-19 patients define multiple targets of vulnerability. *Science* 369, 643–650 (2020). doi:10.1126/science.abc5902 Medline
7. A. Grifoni, D. Weiskopf, S. I. Ramirez, J. Mateus, J. M. Dan, C. R. Moderbacher, S. A. Rawlings, A. Sutherland, L. Premkumar, R. S. Jadi, D. Marrama, A. M. de Silva, A. Frazier, A. F. Carlin, J. A. Greenbaum, B. Peters, F. Krammer, D. M. Smith, S. Crotty, A. Sette, Targets of T Cell Responses to SARS-CoV-2 Coronavirus in Humans with COVID-19 Disease and Unexposed Individuals. *Cell* 181, 1489–1501.e15 (2020). doi:10.1016/j.cell.2020.05.015 Medline
8. G. E. Hartley, E. S. J. Edwards, P. M. Aui, N. Varese, S. Stojanovic, J. McMahon, A. Y. Peleg, I. Boo, H. E. Drummer, P. M. Hogarth, R. E. O'Hehir, M. C. van Zelm, Rapid generation of durable B cell memory to SARS-CoV-2 spike and nucleocapsid

proteins in COVID-19 and convalescence. *Sci. Immunol.* **5**, eabf8891 (2020). doi:10.1126/sciimmunol.abf8891 Medline

9. N. M. A. Okba, M. A. Müller, W. Li, C. Wang, C. H. GeurtsvanKessel, V. M. Corman, M. M. Lamers, R. S. Sikkema, E. de Bruin, F. D. Chandler, Y. Yazdanpanah, Q. Le Hingrat, D. Descamps, N. Houhou-Fidouh, C. B. E. M. Reusken, B. J. Bosch, C. Drosten, M. P. G. Koopmans, B. L. Haagmans, Severe Acute Respiratory Syndrome Coronavirus 2-Specific Antibody Responses in Coronavirus Disease Patients. *Emerg. Infect. Dis.* **26**, 1478–1488 (2020). doi:10.3201/eid2607.200841 Medline
10. A. Sette, S. Crotty, Adaptive immunity to SARS-CoV-2 and COVID-19. *Cell* **184**, 861–880 (2021). doi:10.1016/j.cell.2021.01.007 Medline
11. D. Weiskopf, K. S. Schmitz, M. P. Raadsen, A. Grifoni, N. M. A. Okba, H. Endeman, J. P. C. van den Akker, R. Molenkamp, M. P. G. Koopmans, E. C. M. van Gorp, B. L. Haagmans, R. L. de Swart, A. Sette, R. D. de Vries, Phenotype and kinetics of SARS-CoV-2-specific T cells in COVID-19 patients with acute respiratory distress syndrome. *Sci. Immunol.* **5**, eabd2071 (2020). doi:10.1126/sciimmunol.abd2071 Medline
12. S. S. A. Karim, Vaccines and SARS-CoV-2 variants: The urgent need for a correlate of protection. *Lancet* **397**, 1263–1264 (2021). doi:10.1016/S0140-6736(21)00468-2 Medline
13. F. Krammer, Correlates of protection from SARS-CoV-2 infection. *Lancet* **397**, 1421–1423 (2021). doi:10.1016/S0140-6736(21)00782-0 Medline
14. F. Gong, Y. Dai, T. Zheng, L. Cheng, D. Zhao, H. Wang, M. Liu, H. Pei, T. Jin, D. Yu, P. Zhou, Peripheral CD4+ T cell subsets and antibody response in COVID-19 convalescent individuals. *J. Clin. Invest.* **130**, 6588–6599 (2020). doi:10.1172/JCI141054 Medline
15. A. G. Letizia, Y. Ge, S. Vangeti, C. Goforth, D. L. Weir, N. A. Kuzmina, C. A. Balinsky, H. W. Chen, D. Ewing, A. Soares-Schanoski, M. C. George, W. D. Graham, F. Jones, P. Bharaj, R. A. Lizewski, S. E. Lizewski, J. Marayag, N. Marjanovic, C. M. Miller, S. Mofsowitz, V. D. Nair, E. Nunez, D. M. Parent, C. K. Porter, E. Santa Ana, M. Schilling, D. Stadlbauer, V. A. Sugiharto, M. Termini, P. Sun, R. P. Tracy, F. Krammer, A. Bukreyev, I. Ramos, S. C. Sealfon, SARS-CoV-2 seropositivity and subsequent infection risk in healthy young adults: A prospective cohort study. *Lancet Respir. Med.* S2213-2600(21)00158-2 (2021). doi:10.1016/S2213-2600(21)00158-2 Medline
16. F. Y. Tso, S. J. Lidenge, L. K. Poppe, P. B. Peña, S. R. Privatt, S. J. Bennett, J. R. Ngowi, J. Mwaiselage, M. Belshan, J. A. Siedlik, M. A. Raine, J. B. Ochoa, J. Garcia-Diaz, B. Nossaman, L. Buckner, W. M. Roberts, M. J. Dean, A. C. Ochoa, J. T. West, C. Wood, Presence of antibody-dependent cellular cytotoxicity (ADCC) against SARS-CoV-2 in COVID-19 plasma. *PLOS ONE* **16**, e0247640 (2021). doi:10.1371/journal.pone.0247640 Medline
17. A. Abayasingam, H. Balachandran, D. Agapiou, M. Hammoud, C. Rodrigo, E. Keoshkerian, H. Li, N. A. Brasher, D. Christ, R. Rouet, D. Burnet, B. Grubor-Bauk, W. Rawlinson, S. Turville, A. Aggarwal, A. O. Stella, C. Fichter, F. Brilot, M. Mina, J. J. Post, B. Hudson, N. Gilroy, D. Dwyer, S. C. Sasson, F. Tea, D. Pilli, A. Kelleher, N. Tedla, A. R. Lloyd, M. Martinello, R. A. Bull; COSIN Study Group, Long-term persistence of RBD$^+$ memory B cells encoding neutralizing antibodies in SARS-CoV-2 infection. *Cell Rep Med* **2**, 100228 (2021). doi:10.1016/j.xcrm.2021.100228 Medline
18. J. M. Dan, J. Mateus, Y. Kato, K. M. Hastie, E. D. Yu, C. E. Faliti, A. Grifoni, S. I. Ramirez, S. Haupt, A. Frazier, C. Nakao, V. Rayaprolu, S. A. Rawlings, B. Peters, F. Krammer, V. Simon, E. O. Saphire, D. M. Smith, D. Weiskopf, A. Sette, S. Crotty, Immunological memory to SARS-CoV-2 assessed for up to 8 months after infection. *Science* **371**, eabf4063 (2021). doi:10.1126/science.abf4063 Medline
19. C. Gaebler, Z. Wang, J. C. C. Lorenzi, F. Muecksch, S. Finkin, M. Tokuyama, A. Cho, M. Jankovic, D. Schaefer-Babajew, T. Y. Oliveira, M. Cipolla, C. Viant, C. O. Barnes, Y. Bram, G. Breton, T. Hägglöf, P. Mendoza, A. Hurley, M. Turroja, K. Gordon, K. G. Millard, V. Ramos, F. Schmidt, Y. Weisblum, D. Jha, M. Tankelevich, G. Martinez-Delgado, J. Yee, R. Patel, J. Dizon, C. Unson-O'Brien, I. Shimeliovich, D. F. Robbiani, Z. Zhao, A. Gazumyan, R. E. Schwartz, T. Hatziioannou, P. J. Bjorkman, S. Mehandru, P. D. Bieniasz, M. Caskey, M. C. Nussenzweig, Evolution of antibody immunity to SARS-CoV-2. *Nature* **591**, 639–644 (2021). doi:10.1038/s41586-021-03207-w Medline
20. C. K. Kang, M. Kim, S. Lee, G. Kim, P. G. Choe, W. B. Park, N. J. Kim, C. H. Lee, I. S. Kim, K. Jung, D. S. Lee, H. M. Shin, H. R. Kim, M. D. Oh, Longitudinal Analysis of Human Memory T-Cell Response according to the Severity of Illness up to 8 Months after SARS-CoV-2 Infection. *J. Infect. Dis.* jiab159 (2021). doi:10.1093/infdis/jiab159 Medline
21. J. Y. Noh, J. E. Kwak, J. S. Yang, S. Y. Hwang, J. G. Yoon, H. Seong, H. Hyun, C. S. Lim, S. Y. Yoon, J. Ryou, J. Y. Lee, S. S. Kim, S. H. Park, H. J. Cheong, W. J. Kim, E. C. Shin, J. Y. Song, Longitudinal assessment of anti-SARS-CoV-2 immune responses for six months based on the clinical severity of COVID-19. *J. Infect. Dis.* jiab124 (2021). doi:10.1093/infdis/jiab124 Medline
22. M. Sakharkar, C. G. Rappazzo, W. F. Wieland-Alter, C. L. Hsieh, D. Wrapp, E. S. Esterman, C. I. Kaku, A. Z. Wec, J. C. Geoghegan, J. S. McLellan, R. I. Connor, P. F. Wright, L. M. Walker, Prolonged evolution of the human B cell response to SARS-CoV-2 infection. *Sci. Immunol.* **6**, eabg6916 (2021). doi:10.1126/sciimmunol.abg6916 Medline
23. G. den Hartog, E. R. A. Vos, L. L. van den Hoogen, M. van Boven, R. M. Schepp, G. Smits, J. van Vliet, L. Woudstra, A. J. Wijmenga-Monsuur, C. C. E. van Hagen, E. A. M. Sanders, H. E. de Melker, F. R. M. van der Klis, R. S. van Binnendijk, Persistence of antibodies to SARS-CoV-2 in relation to symptoms in a nationwide prospective study. *Clin. Infect. Dis.* ciab172 (2021). doi:10.1093/cid/ciab172 Medline
24. L. R. Baden, H. M. El Sahly, B. Essink, K. Kotloff, S. Frey, R. Novak, D. Diemert, S. A. Spector, N. Rouphael, C. B. Creech, J. McGettigan, S. Khetan, N. Segall, J. Solis, A. Brosz, C. Fierro, H. Schwartz, K. Neuzil, L. Corey, P. Gilbert, H. Janes, D. Follmann, M. Marovich, J. Mascola, L. Polakowski, J. Ledgerwood, B. S. Graham, H. Bennett, R. Pajon, C. Knightly, B. Leav, W. Deng, H. Zhou, S. Han, M. Ivarsson, J. Miller, T. Zaks; COVE Study Group, Efficacy and Safety of the mRNA-1273 SARS-CoV-2 Vaccine. *N. Engl. J. Med.* **384**, 403–416 (2021). doi:10.1056/NEJMoa2035389 Medline
25. D. Y. Logunov, I. V. Dolzhikova, D. V. Shcheblyakov, A. I. Tukhvatulin, O. V. Zubkova, A. S. Dzharullaeva, A. V. Kovyrshina, N. L. Lubenets, D. M. Grousova, A. S. Erokhova, A. G. Botikov, F. M. Izhaeva, O. Popova, T. A. Ozharovskaya, I. B. Esmagambetov, I. A. Favorskaya, D. I. Zrelkin, D. V. Voronina, D. N. Shcherbinin, A. S. Semikhin, Y. V. Simakova, E. A. Tokarskaya, D. A. Egorova, M. M. Shmarov, N. A. Nikitenko, V. A. Gushchin, E. A. Smolyarchuk, S. K. Zyryanov, S. V. Borisevich, B. S. Naroditsky, A. L. Gintsburg; Gam-COVID-Vac Vaccine Trial Group, Safety and efficacy of an rAd26 and rAd5 vector-based heterologous prime-boost COVID-19 vaccine: An interim analysis of a randomised controlled phase 3 trial in Russia. *Lancet* **397**, 671–681 (2021). doi:10.1016/S0140-6736(21)00234-8 Medline
26. F. P. Polack, S. J. Thomas, N. Kitchin, J. Absalon, A. Gurtman, S. Lockhart, J. L. Perez, G. Pérez Marc, E. D. Moreira, C. Zerbini, R. Bailey, K. A. Swanson, S. Roychoudhury, K. Koury, P. Li, W. V. Kalina, D. Cooper, R. W. Frenck Jr., L. L. Hammitt, Ö. Türeci, H. Nell, A. Schaefer, S. Ünal, D. B. Tresnan, S. Mather, P. R. Dormitzer, U. Şahin, K. U. Jansen, W. C. Gruber; C4591001 Clinical Trial Group, Safety and Efficacy of the BNT162b2 mRNA Covid-19 Vaccine. *N. Engl. J. Med.* **383**, 2603–2615 (2020). doi:10.1056/NEJMoa2034577 Medline
27. M. Voysey, S. A. C. Clemens, S. A. Madhi, L. Y. Weckx, P. M. Folegatti, P. K. Aley, B. Angus, V. L. Baillie, S. L. Barnabas, Q. E. Bhorat, S. Bibi, C. Briner, P. Cicconi, A. M. Collins, R. Colin-Jones, C. L. Cutland, T. C. Darton, K. Dheda, C. J. A. Duncan, K. R. W. Emary, K. J. Ewer, L. Fairlie, S. N. Faust, S. Feng, D. M. Ferreira, A. Finn, A. L. Goodman, C. M. Green, C. A. Green, P. T. Heath, C. Hill, H. Hill, I. Hirsch, S. H. C. Hodgson, A. Izu, S. Jackson, D. Jenkin, C. C. D. Joe, S. Kerridge, A. Koen, G. Kwatra, R. Lazarus, A. M. Lawrie, A. Lelliott, V. Libri, P. J. Lillie, R. Mallory, A. V. A. Mendes, E. P. Milan, A. M. Minassian, A. McGregor, H. Morrison, Y. F. Mujadidi, A. Nana, P. J. O'Reilly, S. D. Padayachee, A. Pittella, E. Plested, K. M. Pollock, M. N. Ramasamy, S. Rhead, A. V. Schwarzbold, N. Singh, A. Smith, R. Song, M. D. Snape, E. Sprinz, R. K. Sutherland, R. Tarrant, E. C. Thomson, M. E. Török, M. Toshner, D. P. J. Turner, J. Vekemans, T. L. Villafana, M. E. E. Watson, C. J. Williams, A. D. Douglas, A. V. S. Hill, T. Lambe, S. C. Gilbert, A. J. Pollard; Oxford COVID Vaccine Trial Group, Safety and efficacy of the ChAdOx1 nCoV-19 vaccine (AZD1222) against SARS-CoV-2: An interim analysis of four randomised controlled trials in Brazil, South Africa, and the UK. *Lancet* **397**, 99–111 (2021). doi:10.1016/S0140-6736(20)32661-1 Medline
28. A. T. Widge, N. G. Rouphael, L. A. Jackson, E. J. Anderson, P. C. Roberts, M. Makhene, J. D. Chappell, M. R. Denison, L. J. Stevens, A. J. Pruijssers, A. B. McDermott, B. Flach, B. C. Lin, N. A. Doria-Rose, S. O'Dell, S. D. Schmidt, K. M. Neuzil, H. Bennett, B. Leav, M. Makowski, J. Albert, K. Cross, V. V. Edara, K. Floyd, M. S. Suthar, W. Buchanan, C. J. Luke, J. E. Ledgerwood, J. R. Mascola, B. S.

Graham, J. H. Beigel; mRNA-1273 Study Group, Durability of Responses after SARS-CoV-2 mRNA-1273 Vaccination. *N. Engl. J. Med.* **384**, 80–82 (2021). doi:10.1056/NEJMc2032195 Medline
29. A. Babiker, C. Marvil, J. J. Waggoner, M. Collins, A. Piantadosi, The Importance and Challenges of Identifying SARS-CoV-2 Reinfections. *J. Clin. Microbiol.* (2020). doi:10.1128/JCM.02769-20 Medline
30. J. S. Lee, S. Y. Kim, T. S. Kim, K. H. Hong, N. H. Ryoo, J. Lee, J. H. Park, S. I. Cho, M. J. Kim, Y. G. Kim, B. Kim, H. S. Shin, H. S. Oh, M. S. Seo, T. R. Gwon, Y. Kim, J. S. Park, B. S. Chin, W. B. Park, S. S. Park, M. W. Seong, Evidence of Severe Acute Respiratory Syndrome Coronavirus 2 Reinfection After Recovery from Mild Coronavirus Disease 2019. *Clin. Infect. Dis.* ciaa1421 (2020). doi:10.1093/cid/ciaa1421 Medline
31. R. L. Tillett, J. R. Sevinsky, P. D. Hartley, H. Kerwin, N. Crawford, A. Gorzalski, C. Laverdure, S. C. Verma, C. C. Rossetto, D. Jackson, M. J. Farrell, S. Van Hooser, M. Pandori, Genomic evidence for reinfection with SARS-CoV-2: A case study. *Lancet Infect. Dis.* **21**, 52–58 (2021). doi:10.1016/S1473-3099(20)30764-7 Medline
32. J. Prévost, A. Finzi, The great escape? SARS-CoV-2 variants evading neutralizing responses. *Cell Host Microbe* **29**, 322–324 (2021). doi:10.1016/j.chom.2021.02.010 Medline
33. A. Rambaut, N. Loman, O. G. Pybus, W. Barclay, J. R. Barrett, A. Carabelli, T. Connor, D. L. Robertson, E. Volz, CoG-UK, Preliminary genomic characterisation of an emergent SARS-CoV-2 lineage in the UK defined by a novel set of spike mutations. *virological.org*, (2020).
34. H. Tegally, E. Wilkinson, M. Giovanetti, A. Iranzadeh, V. Fonseca, J. Giandhari, D. Doolabh, S. Pillay, E. J. San, N. Msomi, K. Mlisana, A. von Gottberg, S. Walaza, M. Allam, A. Ismail, T. Mohale, A. J. Glass, S. Engelbrecht, G. Van Zyl, W. Preiser, F. Petruccione, A. Sigal, D. Hardie, G. Marais, M. Hsiao, S. Korsman, M. A. Davies, L. Tyers, I. Mudau, D. York, C. Maslo, D. Goedhals, S. Abrahams, O. Laguda-Akingba, A. Alisoltani-Dehkordi, A. Godzik, C. K. Wibmer, B. T. Sewell, J. Lourenco, L. C. J. Alcantara, S. L. Kosakovsky Pond, S. Weaver, D. Martin, R. J. Lessells, J. N. Bhiman, C. Williamson, T. de Oliveira, Emergence of a SARS-CoV-2 variant of concern with mutations in spike glycoprotein. *Nature* (2021). doi:10.1038/s41586-021-03402-9
35. E. C. Sabino, L. F. Buss, M. P. S. Carvalho, C. A. Prete Jr., M. A. E. Crispim, N. A. Fraiji, R. H. M. Pereira, K. V. Parag, P. da Silva Peixoto, M. U. G. Kraemer, M. K. Oikawa, T. Salomon, Z. M. Cucunuba, M. C. Castro, A. A. de Souza Santos, V. H. Nascimento, H. S. Pereira, N. M. Ferguson, O. G. Pybus, A. Kucharski, M. P. Busch, C. Dye, N. R. Faria, Resurgence of COVID-19 in Manaus, Brazil, despite high seroprevalence. *Lancet* **397**, 452–455 (2021). doi:10.1016/S0140-6736(21)00183-5 Medline
36. F. Naveca, C. da Costa, V. Nascimento, V. Souza, A. Corado, F. Nascimento, A. Costa, D. Duarte, G. Silva, N. Mejía, K. Pessoa, L. Gonçalves, M. J. Brandão, M. Jesus, R. Pinto, M. Silva, T. Mattos, L. Abdalla, J. Hugo Santos, R. Costa-Filho, G. Luz Wallau, M. Mendonça Siqueira, E. Delatorre, T. Gräf, G. Bello, P. Cristina Resende, SARS-CoV-2 reinfection by the new Variant of Concern (VOC) P.1 in Amazonas, Brazil. *virological.org*, (2021).
37. D. Harrington, B. Kele, S. Pereira, X. Couto-Parada, A. Riddell, S. Forbes, H. Dobbie, T. Cutino-Moguel, Confirmed Reinfection with SARS-CoV-2 Variant VOC-202012/01. *Clin. Infect. Dis.* ciab014 (2021). doi:10.1093/cid/ciab014 Medline
38. N. Zucman, F. Uhel, D. Descamps, D. Roux, J. D. Ricard, Severe reinfection with South African SARS-CoV-2 variant 501Y.V2: A case report. *Clin. Infect. Dis.* ciab129 (2021). doi:10.1093/cid/ciab129 Medline
39. S. A. Madhi, V. Baillie, C. L. Cutland, M. Voysey, A. L. Koen, L. Fairlie, S. D. Padayachee, K. Dheda, S. L. Barnabas, Q. E. Bhorat, C. Briner, G. Kwatra, K. Ahmed, P. Aley, S. Bhikha, J. N. Bhiman, A. E. Bhorat, J. du Plessis, A. Esmail, M. Groenewald, E. Horne, S. H. Hwa, A. Jose, T. Lambe, M. Laubscher, M. Malahleha, M. Masenya, M. Masilela, S. McKenzie, K. Molapo, A. Moultrie, S. Oelofse, F. Patel, S. Pillay, S. Rhead, H. Rodel, L. Rossouw, C. Taoushanis, H. Tegally, A. Thombrayil, S. van Eck, C. K. Wibmer, N. M. Durham, E. J. Kelly, T. L. Villafana, S. Gilbert, A. J. Pollard, T. de Oliveira, P. L. Moore, A. Sigal, A. Izu; NGS-SA Group and the Wits-VIDA COVID Group, Efficacy of the ChAdOx1 nCoV-19 Covid-19 Vaccine against the B.1.351 Variant. *N. Engl. J. Med.* **384**, 1885–1898 (2021). doi:10.1056/NEJMoa2102214 Medline
40. J. Demaret, G. Lefèvre, F. Vuotto, J. Trauet, A. Duhamel, J. Labreuche, P. Varlet, A. Dendooven, S. Stabler, B. Gachet, J. Bauer, B. Prevost, L. Bocket, E. K. Alidjinou, M. Lambert, C. Yelnik, B. Meresse, L. Dubuquoy, D. Launay, S. Dubucquoi, D. Montaigne, E. Woitrain, F. Maggiotto, M. Bou Saleh, I. Top, V. Elsermans, E. Jeanpierre, A. Dupont, S. Susen, T. Brousseau, J. Poissy, K. Faure, M. Labalette; Lille Covid Research Network (LICORNE), Severe SARS-CoV-2 patients develop a higher specific T-cell response. *Clin. Transl. Immunology* **9**, e1217 (2020). doi:10.1002/cti2.1217 Medline
41. D. Schub, V. Klemis, S. Schneitler, J. Mihm, P. M. Lepper, H. Wilkens, R. Bals, H. Eichler, B. C. Gärtner, S. L. Becker, U. Sester, M. Sester, T. Schmidt, High levels of SARS-CoV-2-specific T cells with restricted functionality in severe courses of COVID-19. *JCI Insight* **5**, e142167 (2020). doi:10.1172/jci.insight.142167 Medline
42. R. R. Goel, S. A. Apostolidis, M. M. Painter, D. Mathew, A. Pattekar, O. Kuthuru, S. Gouma, P. Hicks, W. Meng, A. M. Rosenfeld, S. Dysinger, K. A. Lundgreen, L. Kuri-Cervantes, S. Adamski, A. Hicks, S. Korte, D. A. Oldridge, A. E. Baxter, J. R. Giles, M. E. Weirick, C. M. McAllister, J. Dougherty, S. Long, K. D'Andrea, J. T. Hamilton, M. R. Betts, E. T. Luning Prak, P. Bates, S. E. Hensley, A. R. Greenplate, E. J. Wherry, Distinct antibody and memory B cell responses in SARS-CoV-2 naïve and recovered individuals following mRNA vaccination. *Sci. Immunol.* **6**, eabi6950 (2021). doi:10.1126/sciimmunol.abi6950 Medline
43. A. A. M. van der Veldt, S. F. Oosting, A. C. Dingemans, R. S. N. Fehrmann, C. GeurtsvanKessel, M. Jalving, G. F. Rimmelzwaan, P. Kvistborg, C. U. Blank, E. F. Smit, V. E. E. P. Lemmens, T. J. N. Hiltermann, M. P. G. Koopmans, A. L. W. Huckriede, N. Y. Rots, C. A. C. M. van Els, D. van Baarle, J. B. A. G. Haanen, E. G. E. de Vries, COVID-19 vaccination: The VOICE for patients with cancer. *Nat. Med.* **27**, 568–569 (2021). doi:10.1038/s41591-021-01240-w Medline
44. C. H. GeurtsvanKessel, N. M. A. Okba, Z. Igloi, S. Bogers, C. W. E. Embregts, B. M. Laksono, L. Leijten, C. Rokx, B. Rijnders, J. Rahamat-Langendoen, J. P. C. van den Akker, J. J. A. van Kampen, A. A. van der Eijk, R. S. van Binnendijk, B. Haagmans, M. Koopmans, An evaluation of COVID-19 serological assays informs future diagnostics and exposure assessment. *Nat. Commun.* **11**, 3436 (2020). doi:10.1038/s41467-020-17317-y Medline
45. G. den Hartog, R. M. Schepp, M. Kuijer, C. GeurtsvanKessel, J. van Beek, N. Rots, M. P. G. Koopmans, F. R. M. van der Klis, R. S. van Binnendijk, SARS-CoV-2-Specific Antibody Detection for Seroepidemiology: A Multiplex Analysis Approach Accounting for Accurate Seroprevalence. *J. Infect. Dis.* **222**, 1452–1461 (2020). doi:10.1093/infdis/jiaa479 Medline
46. J. J. A. van Kampen, D. A. M. C. van de Vijver, P. L. A. Fraaij, B. L. Haagmans, M. M. Lamers, N. Okba, J. P. C. van den Akker, H. Endeman, D. A. M. P. J. Gommers, J. J. Cornelissen, R. A. S. Hoek, M. M. van der Eerden, D. A. Hesselink, H. J. Metselaar, A. Verbon, J. E. M. de Steenwinkel, G. I. Aron, E. C. M. van Gorp, S. van Boheemen, J. C. Voermans, C. A. B. Boucher, R. Molenkamp, M. P. G. Koopmans, C. Geurtsvankessel, A. A. van der Eijk, Duration and key determinants of infectious virus shedding in hospitalized patients with coronavirus disease-2019 (COVID-19). *Nat. Commun.* **12**, 267 (2021). doi:10.1038/s41467-020-20568-4 Medline
47. Y. Liu, J. Liu, H. Xia, X. Zhang, C. R. Fontes-Garfias, K. A. Swanson, H. Cai, R. Sarkar, W. Chen, M. Cutler, D. Cooper, S. C. Weaver, A. Muik, U. Sahin, K. U. Jansen, X. Xie, P. R. Dormitzer, P. Y. Shi, Neutralizing Activity of BNT162b2-Elicited Serum. *N. Engl. J. Med.* **384**, 1466–1468 (2021). doi:10.1056/NEJMc2102017 Medline
48. D. Planas, T. Bruel, L. Grzelak, F. Guivel-Benhassine, I. Staropoli, F. Porrot, C. Planchais, J. Buchrieser, M. M. Rajah, E. Bishop, M. Albert, F. Donati, M. Prot, S. Behillil, V. Enouf, M. Maquart, M. Smati-Lafarge, E. Varon, F. Schortgen, L. Yahyaoui, M. Gonzalez, J. De Sèze, H. Péré, D. Veyer, A. Sève, E. Simon-Lorière, S. Fafi-Kremer, K. Stefic, H. Mouquet, L. Hocqueloux, S. van der Werf, T. Prazuck, O. Schwartz, Sensitivity of infectious SARS-CoV-2 B.1.1.7 and B.1.351 variants to neutralizing antibodies. *Nat. Med.* **27**, 917–924 (2021). doi:10.1038/s41591-021-01318-5 Medline
49. T. Tada, B. M. Dcosta, M. Samanovic-Golden, R. S. Herati, A. Cornelius, M. J. Mulligan, N. R. Landau, Neutralization of viruses with European, South African, and United States SARS-CoV-2 variant spike proteins by convalescent sera and BNT162b2 mRNA vaccine-elicited antibodies. *bioRxiv* 2021.02.05.430003 (2021). Medline
50. P. Wang, M. S. Nair, L. Liu, S. Iketani, Y. Luo, Y. Guo, M. Wang, J. Yu, B. Zhang, P. D. Kwong, B. S. Graham, J. R. Mascola, J. Y. Chang, M. T. Yin, M. Sobieszczyk, C. A. Kyratsous, L. Shapiro, Z. Sheng, Y. Huang, D. D. Ho, Increased Resistance of SARS-CoV-2 Variants B.1.351 and B.1.1.7 to Antibody Neutralization. *bioRxiv* 2021.01.25.428137 (2021). Medline

51. D. Zhou, W. Dejnirattisai, P. Supasa, C. Liu, A. J. Mentzer, H. M. Ginn, Y. Zhao, H. M. E. Duyvesteyn, A. Tuekprakhon, R. Nutalai, B. Wang, G. C. Paesen, C. Lopez-Camacho, J. Slon-Campos, B. Hallis, N. Coombes, K. Bewley, S. Charlton, T. S. Walter, D. Skelly, S. F. Lumley, C. Dold, R. Levin, T. Dong, A. J. Pollard, J. C. Knight, D. Crook, T. Lambe, E. Clutterbuck, S. Bibi, A. Flaxman, M. Bittaye, S. Belij-Rammerstorfer, S. Gilbert, W. James, M. W. Carroll, P. Klenerman, E. Barnes, S. J. Dunachie, E. E. Fry, J. Mongkolsapaya, J. Ren, D. I. Stuart, G. R. Screaton, Evidence of escape of SARS-CoV-2 variant B.1.351 from natural and vaccine-induced sera. *Cell* **184**, 2348–2361.e6 (2021). doi:10.1016/j.cell.2021.02.037 Medline
52. R. E. Chen, X. Zhang, J. B. Case, E. S. Winkler, Y. Liu, L. A. VanBlargan, J. Liu, J. M. Errico, X. Xie, N. Suryadevara, P. Gilchuk, S. J. Zost, S. Tahan, L. Droit, J. S. Turner, W. Kim, A. J. Schmitz, M. Thapa, D. Wang, A. C. M. Boon, R. M. Presti, J. A. O'Halloran, A. H. J. Kim, P. Deepak, D. Pinto, D. H. Fremont, J. E. Crowe Jr., D. Corti, H. W. Virgin, A. H. Ellebedy, P. Y. Shi, M. S. Diamond, Resistance of SARS-CoV-2 variants to neutralization by monoclonal and serum-derived polyclonal antibodies. *Nat. Med.* **27**, 717–726 (2021). doi:10.1038/s41591-021-01294-w Medline
53. S. Jangra, C. Ye, R. Rathnasinghe, D. Stadlbauer, F. Krammer, V. Simon, L. Martinez-Sobrido, A. Garcia-Sastre, M. Schotsaert, The E484K mutation in the SARS-CoV-2 spike protein reduces but does not abolish neutralizing activity of human convalescent and post-vaccination sera. *medRxiv*, (2021).
54. K. Wu, A. P. Werner, J. I. Moliva, M. Koch, A. Choi, G. B. E. Stewart-Jones, H. Bennett, S. Boyoglu-Barnum, W. Shi, B. S. Graham, A. Carfi, K. S. Corbett, R. A. Seder, D. K. Edwards, mRNA-1273 vaccine induces neutralizing antibodies against spike mutants from global SARS-CoV-2 variants. *bioRxiv* 2021.01.25.427948 (2021). Medline
55. X. Xie, J. Zou, C. R. Fontes-Garfias, H. Xia, K. A. Swanson, M. Cutler, D. Cooper, V. D. Menachery, S. Weaver, P. R. Dormitzer, P. Y. Shi, Neutralization of N501Y mutant SARS-CoV-2 by BNT162b2 vaccine-elicited sera. *bioRxiv* 2021.01.07.425740 (2021). Medline
56. V. V. Edara, C. Norwood, K. Floyd, L. Lai, M. E. Davis-Gardner, W. H. Hudson, G. Mantus, L. E. Nyhoff, M. W. Adelman, R. Fineman, S. Patel, R. Byram, D. N. Gomes, G. Michael, H. Abdullahi, N. Beydoun, B. Panganiban, N. McNair, K. Hellmeister, J. Pitts, J. Winters, J. Kleinhenz, J. Usher, J. B. O'Keefe, A. Piantadosi, J. J. Waggoner, A. Babiker, D. S. Stephens, E. J. Anderson, S. Edupuganti, N. Rouphael, R. Ahmed, J. Wrammert, M. S. Suthar, Infection- and vaccine-induced antibody binding and neutralization of the B.1.351 SARS-CoV-2 variant. *Cell Host Microbe* **29**, 516–521.e3 (2021). doi:10.1016/j.chom.2021.03.009 Medline
57. X. Shen, H. Tang, C. McDanal, K. Wagh, W. Fischer, J. Theiler, H. Yoon, D. Li, B. F. Haynes, K. O. Sanders, S. Gnanakaran, N. Hengartner, R. Pajon, G. Smith, G. M. Glenn, B. Korber, D. C. Montefiori, SARS-CoV-2 variant B.1.1.7 is susceptible to neutralizing antibodies elicited by ancestral spike vaccines. *Cell Host Microbe* **29**, 529–539.e3 (2021). doi:10.1016/j.chom.2021.03.002 Medline
58. W. F. Garcia-Beltran, E. C. Lam, K. St Denis, A. D. Nitido, Z. H. Garcia, B. M. Hauser, J. Feldman, M. N. Pavlovic, D. J. Gregory, M. C. Poznansky, A. Sigal, A. G. Schmidt, A. J. Iafrate, V. Naranbhai, A. B. Balazs, Multiple SARS-CoV-2 variants escape neutralization by vaccine-induced humoral immunity. *Cell* (2021). doi:10.1016/j.cell.2021.03.013
59. Z. Wang, F. Schmidt, Y. Weisblum, F. Muecksch, C. O. Barnes, S. Finkin, D. Schaefer-Babajew, M. Cipolla, C. Gaebler, J. A. Lieberman, T. Y. Oliveira, Z. Yang, M. E. Abernathy, K. E. Huey-Tubman, A. Hurley, M. Turroja, K. A. West, K. Gordon, K. G. Millard, V. Ramos, J. Da Silva, J. Xu, R. A. Colbert, R. Patel, J. Dizon, C. Unson-O'Brien, I. Shimeliovich, A. Gazumyan, M. Caskey, P. J. Bjorkman, R. Casellas, T. Hatziioannou, P. D. Bieniasz, M. C. Nussenzweig, mRNA vaccine-elicited antibodies to SARS-CoV-2 and circulating variants. *Nature* **592**, 616–622 (2021). doi:10.1038/s41586-021-03324-6 Medline
60. P. Bruhns, B. Iannascoli, P. England, D. A. Mancardi, N. Fernandez, S. Jorieux, M. Daëron, Specificity and affinity of human Fcgamma receptors and their polymorphic variants for human IgG subclasses. *Blood* **113**, 3716–3725 (2009). doi:10.1182/blood-2008-09-179754 Medline
61. R. D. de Vries, N. J. Nieuwkoop, M. Pronk, E. de Bruin, G. Leroux-Roels, E. G. W. Huijskens, R. S. van Binnendijk, F. Krammer, M. P. G. Koopmans, G. F. Rimmelzwaan, Influenza virus-specific antibody dependent cellular cytoxicity induced by vaccination or natural infection. *Vaccine* **35**, 238–247 (2017). doi:10.1016/j.vaccine.2016.11.082 Medline
62. R. D. de Vries, N. J. Nieuwkoop, F. R. M. van der Klis, M. P. G. Koopmans, F. Krammer, G. F. Rimmelzwaan, Primary Human Influenza B Virus Infection Induces Cross-Lineage Hemagglutinin Stalk-Specific Antibodies Mediating Antibody-Dependent Cellular Cytoxicity. *J. Infect. Dis.* **217**, 3–11 (2017). doi:10.1093/infdis/jix546 Medline
63. S. Jegaskanda, K. L. Laurie, T. H. Amarasena, W. R. Winnall, M. Kramski, R. De Rose, I. G. Barr, A. G. Brooks, P. C. Reading, S. J. Kent, Age-associated cross-reactive antibody-dependent cellular cytotoxicity toward 2009 pandemic influenza A virus subtype H1N1. *J. Infect. Dis.* **208**, 1051–1061 (2013). doi:10.1093/infdis/jit294 Medline
64. M. Zheng, Y. Gao, G. Wang, G. Song, S. Liu, D. Sun, Y. Xu, Z. Tian, Functional exhaustion of antiviral lymphocytes in COVID-19 patients. *Cell. Mol. Immunol.* **17**, 533–535 (2020). doi:10.1038/s41423-020-0402-2 Medline
65. A. J. Wilk, A. Rustagi, N. Q. Zhao, J. Roque, G. J. Martínez-Colón, J. L. McKechnie, G. T. Ivison, T. Ranganath, R. Vergara, T. Hollis, L. J. Simpson, P. Grant, A. Subramanian, A. J. Rogers, C. A. Blish, A single-cell atlas of the peripheral immune response in patients with severe COVID-19. *Nat. Med.* **26**, 1070–1076 (2020). doi:10.1038/s41591-020-0944-y Medline
66. C. Huang, Y. Wang, X. Li, L. Ren, J. Zhao, Y. Hu, L. Zhang, G. Fan, J. Xu, X. Gu, Z. Cheng, T. Yu, J. Xia, Y. Wei, W. Wu, X. Xie, W. Yin, H. Li, M. Liu, Y. Xiao, H. Gao, L. Guo, J. Xie, G. Wang, R. Jiang, Z. Gao, Q. Jin, J. Wang, B. Cao, Clinical features of patients infected with 2019 novel coronavirus in Wuhan, China. *Lancet* **395**, 497–506 (2020). doi:10.1016/S0140-6736(20)30183-5 Medline
67. J. Dufloo, L. Grzelak, I. Staropoli, Y. Madec, L. Tondeur, A. Francois, S. Pelleau, A. Wiedemann, C. Planchais, J. Buchrieser, R. Robinot, M. Ungeheuer, H. Mouquet, P. Charneau, M. White, Y. Levy, B. Hoen, A. Fontanet, O. Schwartz, T. Bruel, Asymptomatic and symptomatic SARS-CoV-2 infections elicit polyfunctional antibodies. *medRxiv*, (2020).
68. M. D. Larsen, E. L. de Graaf, M. E. Sonneveld, H. R. Plomp, J. Nouta, W. Hoepel, H. J. Chen, F. Linty, R. Visser, M. Brinkhaus, T. Šuštić, S. W. de Taeye, A. E. H. Bentlage, S. Toivonen, C. A. M. Koeleman, S. Sainio, N. A. Kootstra, P. J. M. Brouwer, C. E. Geyer, N. I. L. Derksen, G. Wolbink, M. de Winther, R. W. Sanders, M. J. van Gils, S. de Bruin, A. P. J. Vlaar, T. Rispens, J. den Dunnen, H. L. Zaaijer, M. Wuhrer, C. Ellen van der Schoot, G. Vidarsson; Amsterdam UMC COVID-19; biobank study group, Afucosylated IgG characterizes enveloped viral responses and correlates with COVID-19 severity. *Science* **371**, eabc8378 (2021). doi:10.1126/science.abc8378 Medline
69. A. D. Redd, A. Nardin, H. Kared, E. M. Bloch, A. Pekosz, O. Laeyendecker, B. Abel, M. Fehlings, T. C. Quinn, A. A. Tobian, CD8+ T cell responses in COVID-19 convalescent individuals target conserved epitopes from multiple prominent SARS-CoV-2 circulating variants. *medRxiv*, (2021).
70. B. Agerer, M. Koblischke, V. Gudipati, L. F. Montaño-Gutierrez, M. Smyth, A. Popa, J. W. Genger, L. Endler, D. M. Florian, V. Mühlgrabner, M. Graninger, S. W. Aberle, A. M. Husa, L. E. Shaw, A. Lercher, P. Gattinger, R. Torralba-Gombau, D. Trapin, T. Penz, D. Barreca, I. Fae, S. Wenda, M. Traugott, G. Walder, W. F. Pickl, V. Thiel, F. Allerberger, H. Stockinger, E. Puchhammer-Stöckl, W. Weninger, G. Fischer, W. Hoepler, E. Pawelka, A. Zoufaly, R. Valenta, C. Bock, W. Paster, R. Geyeregger, M. Farlik, F. Halbritter, J. B. Huppa, J. H. Aberle, A. Bergthaler, SARS-CoV-2 mutations in MHC-I-restricted epitopes evade $CD8^+$ T cell responses. *Sci. Immunol.* **6**, eabg6461 (2021). doi:10.1126/sciimmunol.abg6461 Medline
71. A. Tarke, J. Sidney, N. Methot, Y. Zhang, J. M. Dan, B. Goodwin, P. Rubiro, A. Sutherland, R. da Silva Antunes, A. Frazier, S. A. Rawlings, D. M. Smith, B. Peters, R. H. Scheuermann, D. Weiskopf, S. Crotty, A. Grifoni, A. Sette, Negligible impact of SARS-CoV-2 variants on $CD4^+$ and $CD8^+$ T cell reactivity in COVID-19 exposed donors and vaccinees. *bioRxiv* 2021.02.27.433180 (2021). Medline
72. J. Mateus, A. Grifoni, A. Tarke, J. Sidney, S. I. Ramirez, J. M. Dan, Z. C. Burger, S. A. Rawlings, D. M. Smith, E. Phillips, S. Mallal, M. Lammers, P. Rubiro, L. Quiambao, A. Sutherland, E. D. Yu, R. da Silva Antunes, J. Greenbaum, A. Frazier, A. J. Markmann, L. Premkumar, A. de Silva, B. Peters, S. Crotty, A. Sette, D. Weiskopf, Selective and cross-reactive SARS-CoV-2 T cell epitopes in unexposed humans. *Science* **370**, 89–94 (2020). doi:10.1126/science.abd3871 Medline
73. I. Cassaniti, E. Percivalle, F. Bergami, A. Piralla, G. Comolli, R. Bruno, M. Vecchia, M. Sambo, M. Colaneri, V. Zuccaro, M. Benazzo, C. Robotti, A. Calastri, E. Maiorano, A. Ferrari, G. Cambiè, F. Baldanti, SARS-CoV-2 specific T-cell immunity in COVID-19 convalescent patients and unexposed controls measured by ex vivo

ELISpot assay. *Clin. Microbiol. Infect.* S1198-743X(21)00145-2 (2021). doi:10.1016/j.cmi.2021.03.010 Medline

74. A. Ogbe, B. Kronsteiner, D. T. Skelly, M. Pace, A. Brown, E. Adland, K. Adair, H. D. Akhter, M. Ali, S. E. Ali, A. Angyal, M. A. Ansari, C. V. Arancibia-Cárcamo, H. Brown, S. Chinnakannan, C. Conlon, C. de Lara, T. de Silva, C. Dold, T. Dong, T. Donnison, D. Eyre, A. Flaxman, H. Fletcher, J. Gardner, J. T. Grist, C. P. Hackstein, K. Jaruthamsophon, K. Jeffery, T. Lambe, L. Lee, W. Li, N. Lim, P. C. Matthews, A. J. Mentzer, S. C. Moore, D. J. Naisbitt, M. Ogese, G. Ogg, P. Openshaw, M. Pirmohamed, A. J. Pollard, N. Ramamurthy, P. Rongkard, S. Rowland-Jones, O. Sampson, G. Screaton, A. Sette, L. Stafford, C. Thompson, P. J. Thomson, R. Thwaites, V. Vieira, D. Weiskopf, P. Zacharopoulou, L. Turtle, P. Klenerman, P. Goulder, J. Frater, E. Barnes, S. Dunachie; Oxford Immunology Network Covid-19 Response T Cell Consortium; Oxford Protective T Cell Immunology for COVID-19 (OPTIC) Clinical Team, T cell assays differentiate clinical and subclinical SARS-CoV-2 infections from cross-reactive antiviral responses. *Nat. Commun.* **12**, 2055 (2021). doi:10.1038/s41467-021-21856-3 Medline
75. U. Sahin, A. Muik, E. Derhovanessian, I. Vogler, L. M. Kranz, M. Vormehr, A. Baum, K. Pascal, J. Quandt, D. Maurus, S. Brachtendorf, V. Lörks, J. Sikorski, R. Hilker, D. Becker, A. K. Eller, J. Grützner, C. Boesler, C. Rosenbaum, M. C. Kühnle, U. Luxemburger, A. Kemmer-Brück, D. Langer, M. Bexon, S. Bolte, K. Karikó, T. Palanche, B. Fischer, A. Schultz, P. Y. Shi, C. Fontes-Garfias, J. L. Perez, K. A. Swanson, J. Loschko, I. L. Scully, M. Cutler, W. Kalina, C. A. Kyratsous, D. Cooper, P. R. Dormitzer, K. U. Jansen, Ö. Türeci, COVID-19 vaccine BNT162b1 elicits human antibody and T_H1 T cell responses. *Nature* **586**, 594–599 (2020). doi:10.1038/s41586-020-2814-7 Medline
76. M. McCallum, J. Bassi, A. Marco, A. Chen, A. C. Walls, J. D. Iulio, M. A. Tortorici, M. J. Navarro, C. Silacci-Fregni, C. Saliba, M. Agostini, D. Pinto, K. Culap, S. Bianchi, S. Jaconi, E. Cameroni, J. E. Bowen, S. W. Tilles, M. S. Pizzuto, S. B. Guastalla, G. Bona, A. F. Pellanda, C. Garzoni, W. C. Van Voorhis, L. E. Rosen, G. Snell, A. Telenti, H. W. Virgin, L. Piccoli, D. Corti, D. Veesler, SARS-CoV-2 immune evasion by variant B.1.427/B.1.429. *bioRxiv* 2021.03.31.437925 (2021). Medline
77. M. Hoffmann, P. Arora, R. Groß, A. Seidel, B. F. Hörnich, A. S. Hahn, N. Krüger, L. Graichen, H. Hofmann-Winkler, A. Kempf, M. S. Winkler, S. Schulz, H. M. Jäck, B. Jahrsdörfer, H. Schrezenmeier, M. Müller, A. Kleger, J. Münch, S. Pöhlmann, SARS-CoV-2 variants B.1.351 and P.1 escape from neutralizing antibodies. *Cell* **184**, 2384–2393.e12 (2021). doi:10.1016/j.cell.2021.03.036 Medline
78. A. Bal, G. Destras, A. Gaymard, K. Stefic, J. Marlet, S. Eymieux, H. Regue, Q. Semanas, C. d'Aubarede, G. Billaud, F. Laurent, C. Gonzalez, Y. Mekki, M. Valette, M. Bouscambert, C. Gaudy-Graffin, B. Lina, F. Morfin, L. Josset; COVID-Diagnosis HCL Study Group, Two-step strategy for the identification of SARS-CoV-2 variant of concern 202012/01 and other variants with spike deletion H69-V70, France, August to December 2020. *Euro Surveill.* **26**, ••• (2021). doi:10.2807/1560-7917.ES.2021.26.3.2100008 Medline
79. J. H. Gong, G. Maki, H. G. Klingemann, Characterization of a human cell line (NK-92) with phenotypical and functional characteristics of activated natural killer cells. *Leukemia* **8**, 652–658 (1994). Medline
80. R. N. Kirchdoerfer, C. A. Cottrell, N. Wang, J. Pallesen, H. M. Yassine, H. L. Turner, K. S. Corbett, B. S. Graham, J. S. McLellan, A. B. Ward, Pre-fusion structure of a human coronavirus spike protein. *Nature* **531**, 118–121 (2016). doi:10.1038/nature17200 Medline

Acknowledgments: The authors would like to thank Amber Weevers, Sandra Scherbeijn, Faye de Wilt, and the Erasmus MC ARBO swab unit and call center for excellent assistance. **Funding:** This work was financially supported by the Top Sector Life Sciences & Health; Health~Holland (grant EMCLHS20017: DG, LG. RDdV), the European Union's Horizon 2020 research and innovation program under grant No. 101003589 (RECoVER: BBOM, MPGK), ZonMW (grant agreement No. 10150062010005: BBOM, MPGK; and No. 10150062010008: BLH), the Netherlands Organization for Scientific Research (NWO) Vici grant (RWS), the Bill & Melinda Gates Foundation grant INV-002022 (RWS), and an Amsterdam UMC AMC Fellowship (MJvG). Author contributions: Conceptualization RDdV, CHGvK Formal Analysis DG, MCS, RDdV, CHGvK Funding acquisition MJvG, RWS, BLH, RLdS, MPGK, RSvB, RDdV, CHGvK Investigation DG, MCS, SB, GdH, LG, NNN, KSS, LCR, GS, AC, DvM, BBOM, RM, RSvB, RDdV Project administration SB, JATvO, ED, RDdV, CHGvK Resources TGC, MJvG, RWS, HJdJ, BLH, RDdV, CHGvK Supervision RWS, BLH, RLdS, MPGK, RSvB, RDdV, CHGvK Visualization: DG, MCS, GdH, RSvB, RDdV, CHGvK Writing – original draft: DG, MCS, RDdV, CHGvK Writing – review and editing All authors reviewed and edited the final version Competing interests: The authors declare that they have no competing interests. Data and materials availability: All data are available in the main text or the supplementary materials. This work is licensed under a Creative Commons Attribution 4.0 International (CC BY 4.0) license, which permits unrestricted use, distribution, and reproduction in any medium, provided the original work is properly cited. To view a copy of this license, visit https://creativecommons.org/licenses/by/4.0/. This license does not apply to figures/photos/artwork or other content included in the article that is credited to a third party; obtain authorization from the rights holder before using such material.

Submitted 26 April 2021
Accepted 20 May 2021
Published First Release 25 May 2021
10.1126/sciimmunol.abj1750

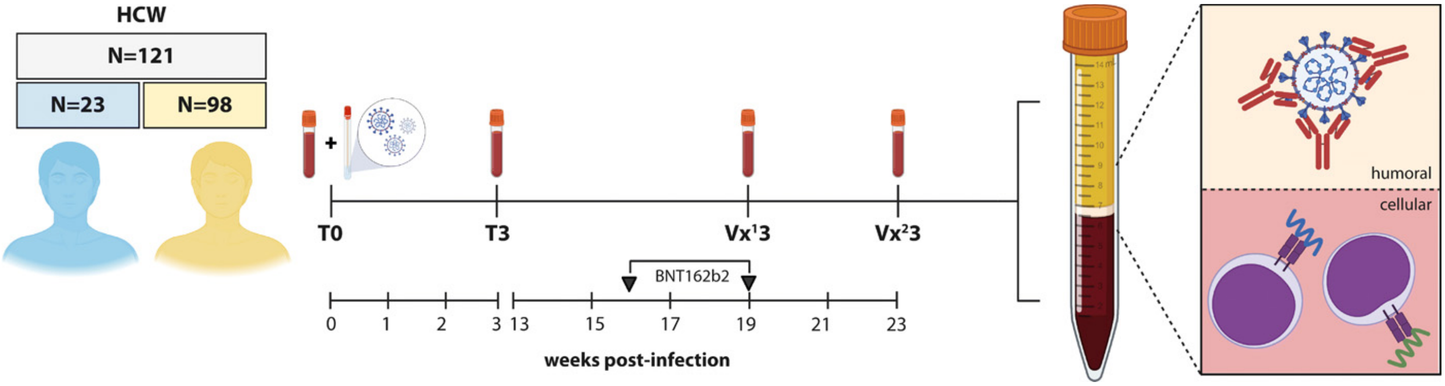

Fig. 1. HCW study design. N=121 HCW were enrolled in a prospective SARS-CoV-2 infection and vaccination study. Upon symptomatic presentation to occupational health services a paired nasopharyngeal swab and EDTA blood sample was obtained (T0). A second EDTA blood sample was obtained 3 weeks after diagnostic RT-PCR (T3). Based on the diagnostic RT-PCR result at T0 and serology result at T3, 98 COVID-19 naive (yellow) and 23 COVID-19 recovered (blue) HCW were enrolled in the vaccination study on average 50 days after inclusion. N=13 COVID-19 recovered and N=12 COVID-19 naive participants were randomly selected for in-depth analysis. Blood samples were collected after the first ($Vx^1 3$) and second ($Vx^2 3$) vaccination, processed and subsequently used for downstream serological and cellular assays.

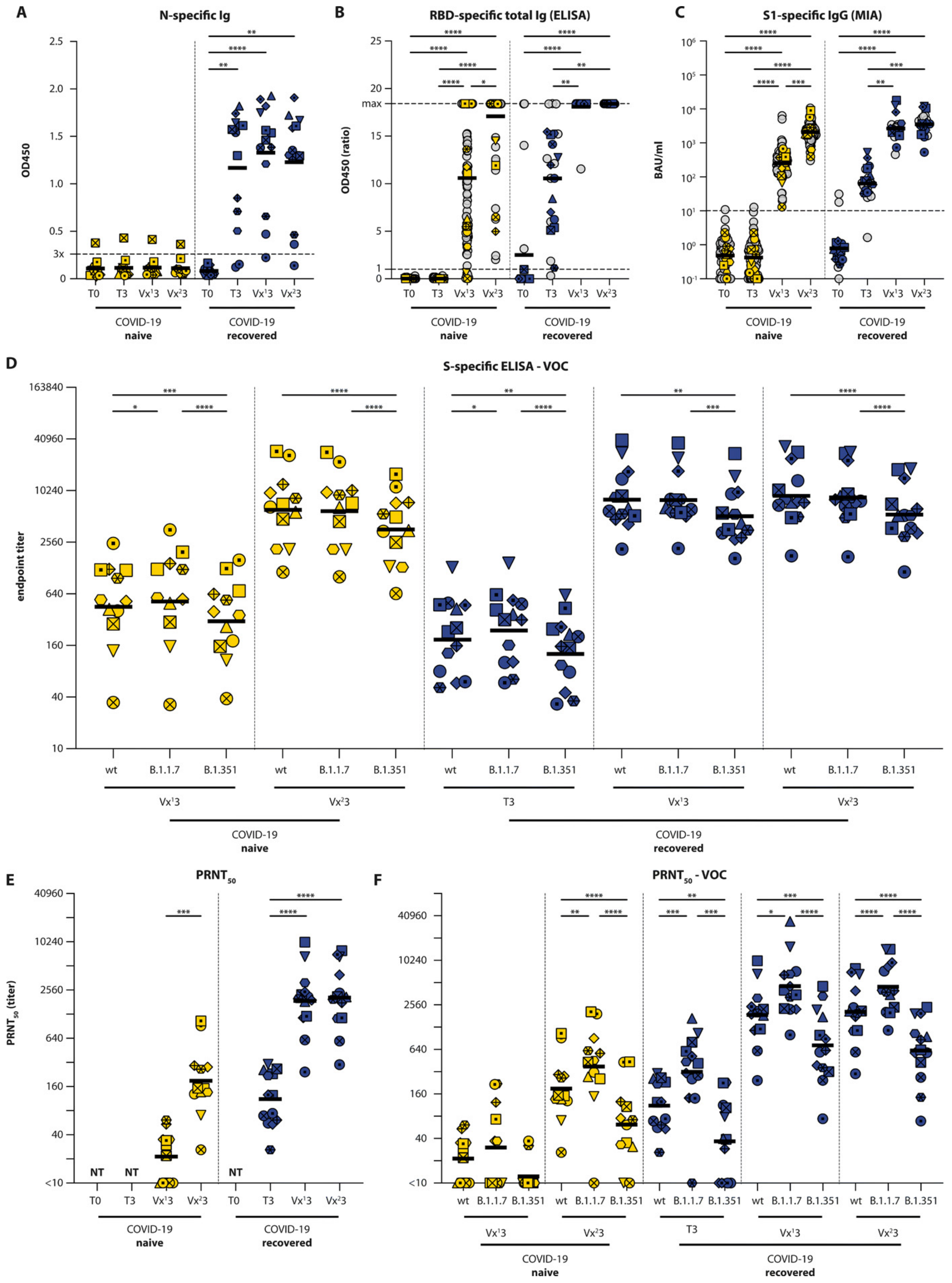
A
N-specific Ig
OD450
T0 T3 Vx¹3 Vx²3 T0 T3 Vx¹3 Vx²3
COVID-19 naive
COVID-19 recovered
B
RBD-specific total Ig (ELISA)
OD450 (ratio)
max
C
S1-specific IgG (MIA)
BAU/ml
D
S-specific ELISA - VOC
endpoint titer
wt B.1.1.7 B.1.351
Vx¹3 Vx²3 T3 Vx¹3 Vx²3
COVID-19 naive
COVID-19 recovered
E
PRNT50
PRNT50 (titer)
NT
F
PRNT50 - VOC

Fig. 2. Detection of SARS-CoV-2-specific humoral responses. Total immunoglobulin levels were measured in COVID-19 naive (yellow) and recovered (blue) donors at the acute, convalescent, post-vaccination 1 and post-vaccination 2 stage (T0, T3, $Vx^1 3$, $Vx^2 3$) by an (**A**) ELISA against nucleocapsid (N) and (**B**) receptor-binding domain (RBD). (**C**) Quantitative IgG against S1 was measured by a Luminex bead assay. (**D**) Antibody binding to WT SARS-CoV-2 and VOC B.1.1.7 and B.1.351 was determined by endpoint titration in ELISA. Virus neutralization was measured by $PRNT_{50}$ against (**E**) WT SARS-CoV-2 (D614G) and (**F**) VOC. Analyses in panel B and C were performed on 121 participants, in-depth analyses in panel A, D, E, F on 25 participants. Timepoints in panel A, B and C were compared by performing a non-parametric repeated measures Friedman test. Endpoint titers between VOC in panel D were compared by RM one-way ANOVA or Friedman test. $PRNT_{50}$ titers in panel D and E were compared by RM one-way ANOVA. * $p < 0.05$, ** $p < 0.01$, *** $p < 0.001$, **** $p < 0.0001$. Symbol shapes indicate individual donors and are consistent throughout the figures. Lines in panel A and B show the mean, lines in panel C, D, E and F show geometric means. Dotted lines represent cut-off values for positivity (3x background OD450 in A, OD450 ratio = 1 in B, 10,08 BAU/ml in C). NT: not tested.

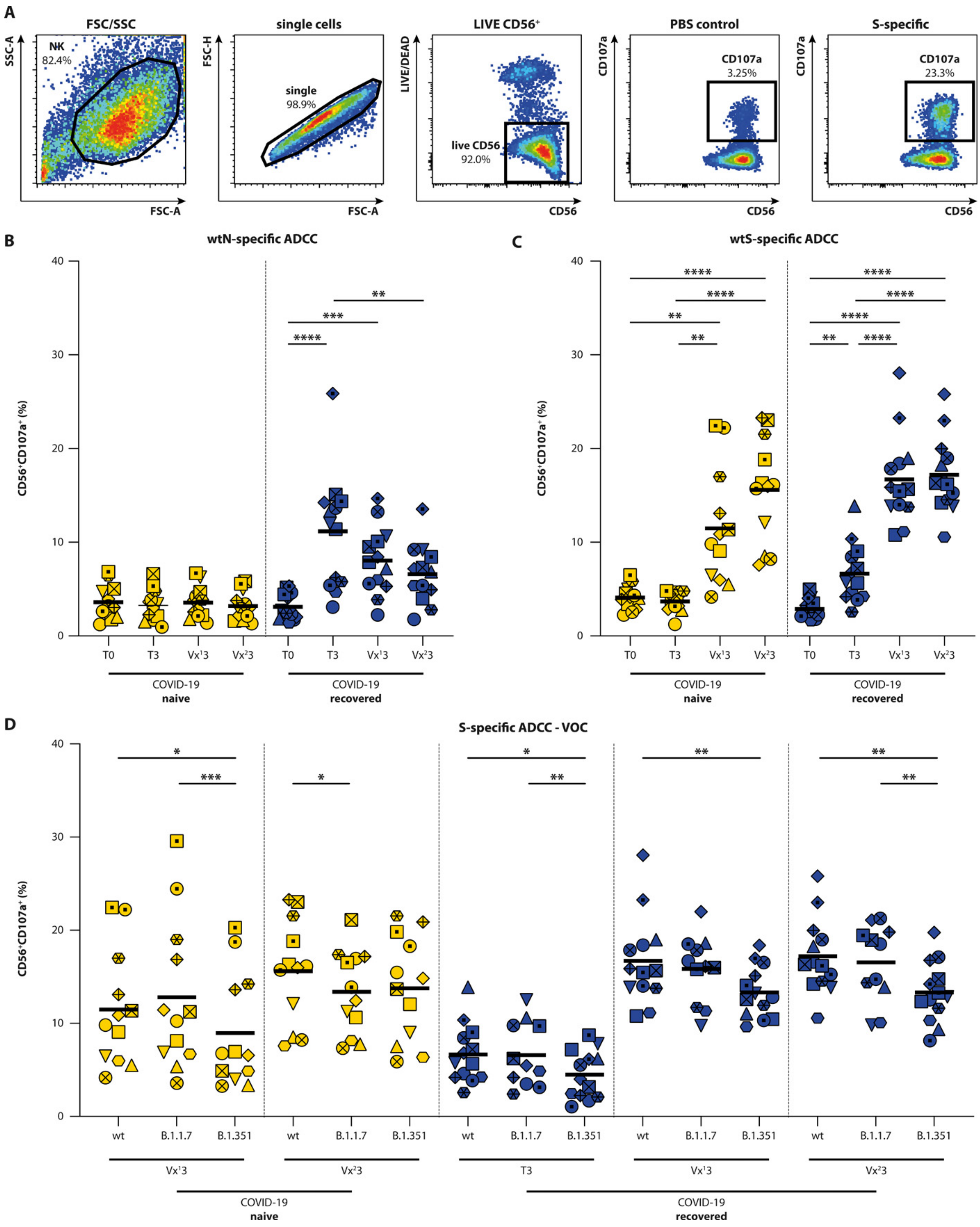
A
FSC/SSC
NK
82.4%
SSC-A
FSC-A
single cells
single
98.9%
FSC-H
FSC-A
LIVE CD56+
live CD56
92.0%
LIVE/DEAD
CD56
PBS control
CD107a
3.25%
CD107a
CD56
S-specific
CD107a
23.3%
CD107a
CD56
B
wtN-specific ADCC
CD56+CD107a+ (%)
T0
T3
Vx13
Vx23
COVID-19
naive
COVID-19
recovered
C
wtS-specific ADCC
D
S-specific ADCC - VOC
wt
B.1.1.7
B.1.351
Vx13
Vx23
T3
COVID-19
naive
COVID-19
recovered

Fig. 3. Detection of ADCC-mediating antibodies by measuring NK92.05 degranulation. (**A**) Gating strategy for detection of degranulating NK cells: (1) NK92.05-CD16 cells are selected on basis of size and granularity, (2) exclusion of doublets, and (3) selection of LIVE and $CD56^{+}$ cells. Degranulation is measured as percentage $CD107a^{+}$ cells within the NK fraction, PBS coating is included as background control. (**B-C**) ADCC-mediating antibodies were detected in COVID-19 naive (yellow) and recovered (blue) donors at the acute, convalescent, post-vaccination 1 and post-vaccination 2 stage (T0, T3, $Vx^{1}3$, $Vx^{2}3$) against the WT N (**B**) and S (**C**) protein. (**D**) ADCC-mediating antibody reactivity with WT SARS-CoV-2 and VOC B.1.1.7 and B.1.351. These analyses were performed on 25 participants. Timepoints in panel B and C were compared by performing a non-parametric repeated measures Friedman test. Differences between variants were assessed by mixed-effect models. * $p < 0.05$, ** $p < 0.01$, *** $p < 0.001$, **** $p < 0.0001$. Symbol shapes indicate individual donors and are consistent throughout the figures. Lines indicate mean responses.

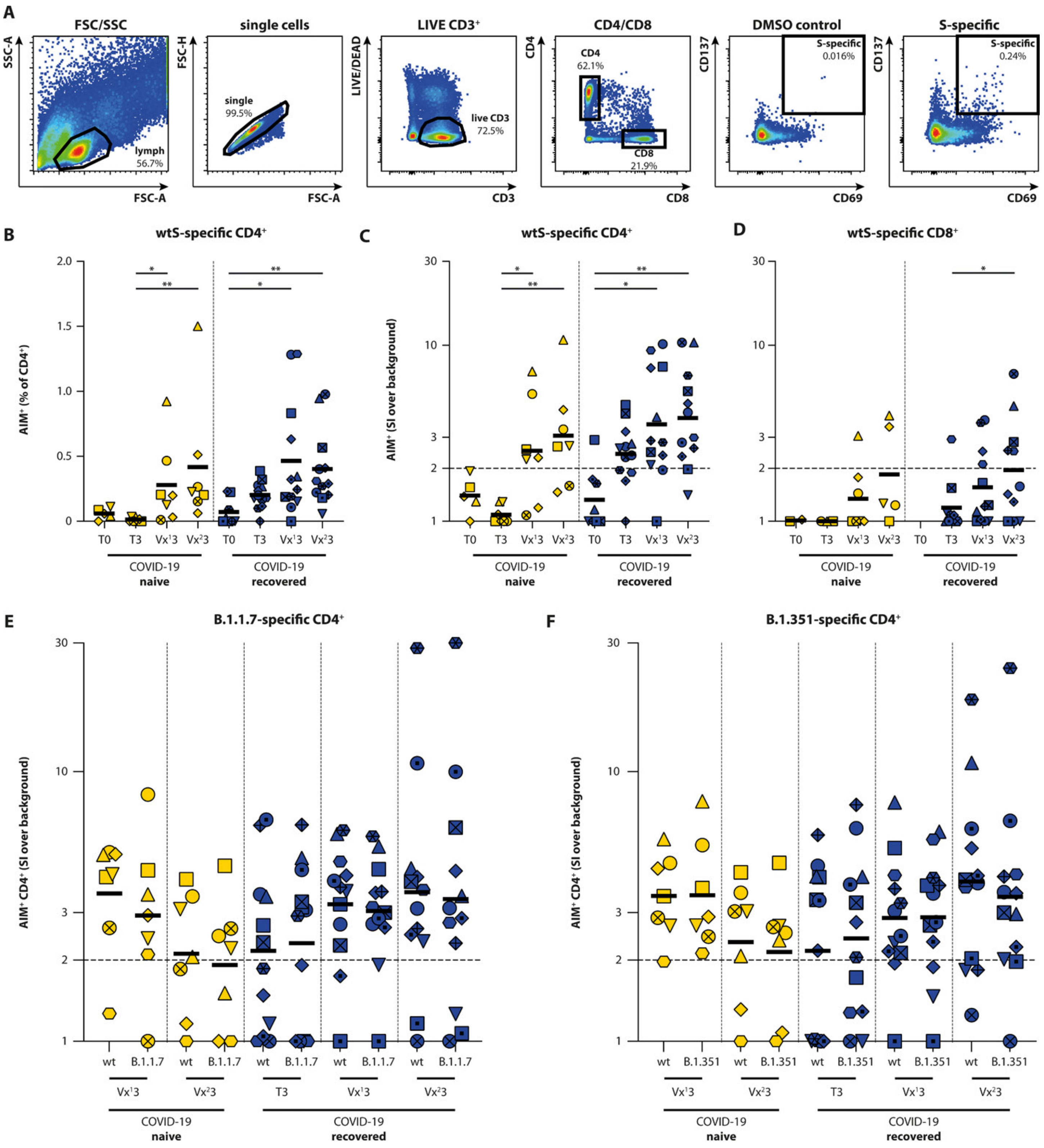
A
FSC/SSC
single cells
LIVE CD3+
CD4/CD8
DMSO control
S-specific
lymph 56.7%
single 99.5%
live CD3 72.5%
CD4 62.1%
CD8 21.9%
S-specific 0.016%
S-specific 0.24%
B
wtS-specific CD4+
AIM+ (% of CD4+)
C
wtS-specific CD4+
AIM+ (SI over background)
D
wtS-specific CD8+
T0
T3
Vx¹3
Vx²3
COVID-19 naive
COVID-19 recovered
E
B.1.1.7-specific CD4+
AIM+ CD4+ (SI over background)
wt
B.1.1.7
F
B.1.351-specific CD4+
B.1.351

Fig. 4. Detection of S-specific T cells by measuring up-regulation of activation-induced markers (AIM). (**A**) Gating strategy for virus-specific T cells cells that up-regulate AIM: (1) Lymphocytes are selected on basis of size and granularity, (2) exclusion of doublets, (3) selection of LIVE and $CD3^+$ cells, and (4) division into $CD4^+$ and $CD8^+$ T cells. Activation is measured as percentage $CD69^+$/$CD137^+$ double-positive cells within the CD4 or CD8 fraction, DMSO stimulation is included as background control. (**B, C, D**) Antigen specific activation of $CD4^+$ and $CD8^+$ T cells in COVID-19 naive (yellow) and COVID-19 recovered (blue) donors at the acute, convalescent, post-vaccination 1 and post-vaccination 2 stage (T0, T3, $Vx^1 3$, $Vx^2 3$) by overlapping peptide pools covering the full WT S protein. Activation of SARS-CoV-2 specific $CD4^+$ T cells is shown as percentage AIM^+ cells within the $CD4^+$ subset after (**B**) subtraction of the DMSO background or (**C**) as a stimulation index (SI) by dividing specific activation over background activation. (**D**) Activation of SARS-CoV-2-specific $CD8^+$ T cells is shown as SI. An SI-index of 2 or higher is considered a positive T-cell response. (**E-F**) Antigen-specific activation of $CD4^+$ T cells by peptide pools exclusively covering mutational regions in VOC B.1.1.7 and B.1.351, compared against homologous WT peptide pools. Antigen-specific T-cell responses are shown as SI. These analyses were performed in 20 participants. Timepoints in panel B, C and D were compared by performing a Kruskal-Wallis test. Differences between variants were compared by performing Wilcoxon test. * $p < 0.05$, ** $p < 0.01$, *** $p < 0.001$, **** $p < 0.0001$. Symbol shapes indicate individual donors and are consistent throughout the figures. Lines indicate mean (B) or geometric mean (C, D, E, F) responses. Low cell count samples (<10,000 or <5,000 events within $CD4^+$ or $CD8^+$ gate, respectively) were excluded.

Table 1. Characteristics of study participants prior to vaccination.

				In-depth analysis	
	**All**	**COVID-19 Recovered**	**COVID-19 Naive**	**COVID-19 Recovered**	**COVID-19 Naive**
**N**	121	23	98	13	12
**Gender**					
Male	39 (32.2%)	6 (26.1%)	33 (35.6%)	3 (23%)	1 (8.3%)
Female	82 (67.8%)	17 (73.9%)	65 (66.3%)	10 (77%)	11 (91.7%)
**Age**	40	42	38.5	42	47
(median + Q1-Q3)	(34.8-55.8)	(34.5-56.5)	(34.3-57.3)	(34.5-56.5)	(34.3-55.0)
< 30	21 (17.4%)	4 (17.4%)	17 (17.3%)	3 (23.1%)	1 (8.3%)
30-44	56 (46.3%)	10 (43.5%)	46 (46.9%)	5 (38.5%)	5 (41.7%)
45-59	35 (28.9%)	7 (30.4%)	28 (28.6%)	4 (30.8%)	5 (41.7%)
> 60	9 (7.4%)	2 (8.7%)	7 (7.1%)	1 (7.7%)	1 (8.3%)
**Days between diagnosis and vaccination** (median)	-	54	-	47	-
< 30 days		2 (8.7%)		2 (15.4%)	
30-60 days		12 (52.2%)		10 (85.6%)	
>60 days		9 (39.1%)		1 (7.7%)	
