# Supplementary material for: SARS-CoV-2 variants of concern partially escape humoral but not T cell responses in COVID-19 convalescent donors and vaccine recipients
Source: Sci Immunol. 2021 May 25;6(59):eabj1750. doi: 10.1126/sciimmunol.abj1750 (PMC9268159; doi:10.1126/sciimmunol.abj1750)
Supplement: Supplementary file 2 — Figs. S1 to S5 Tables S1 to S3 [file sciimmunol.abj1750_sm.pdf]

Science Immunology
AAAS

Supplementary Materials for

SARS-CoV-2 variants of concern partially escape humoral but not T cell responses in COVID-19 convalescent donors and vaccine recipients

Daryl Geers *et al.*

Corresponding author: Rory D. de Vries, r.d.devries@erasmusmc.nl; Corine H. GeurtsvanKessel, c.geurtsvankessel@erasmusmc.nl

Sci. Immunol. **6**, eabj1750 (2021)
DOI: 10.1126/sciimmunol.abj1750

The PDF file includes:

Figs. S1 to S5
Tables S1 to S3

Other Supplementary Material for this manuscript includes the following:

Data file S1

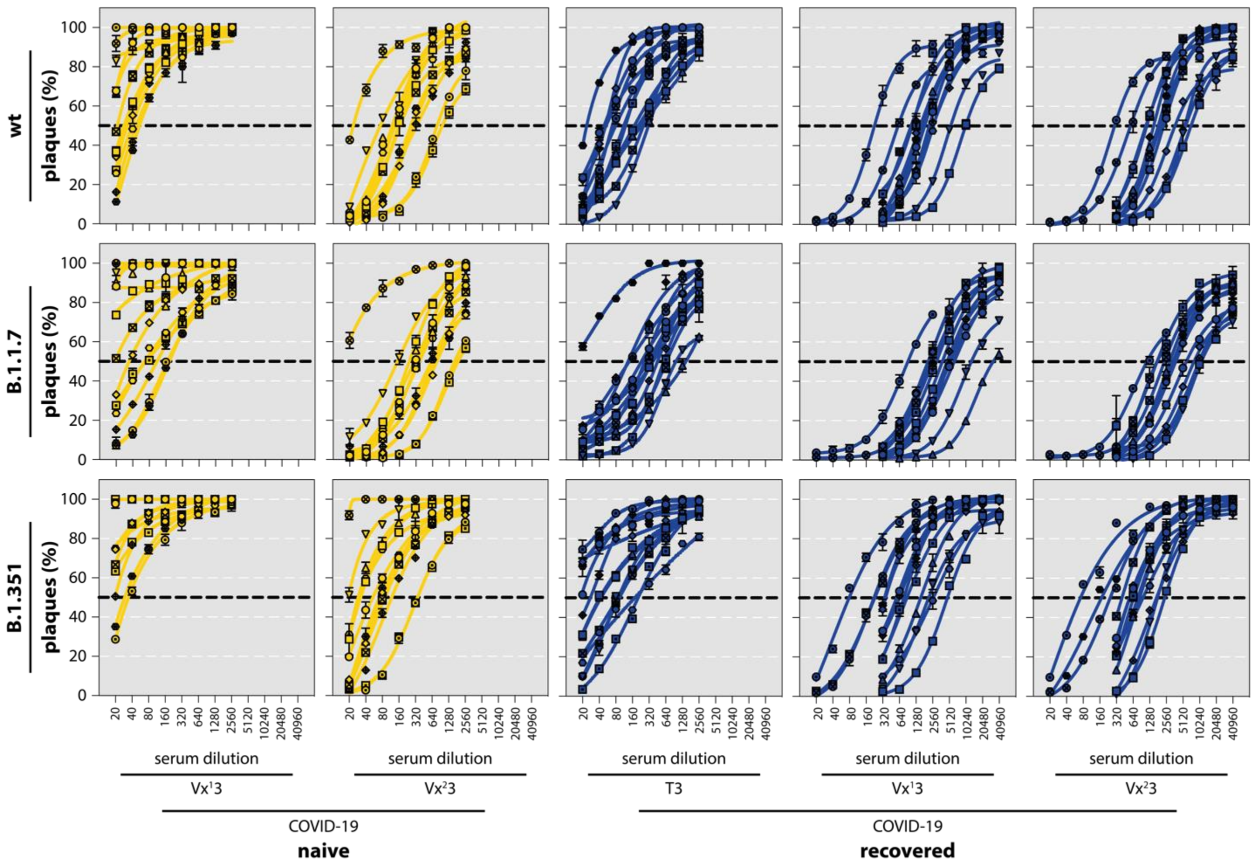

Figure S1. Normalized S-curves for determining $PRNT_{50}$. Sera were 2-fold diluted starting at a dilution of 1:10 in duplicate, followed by adding virus suspension leading to ±1,000 plaques/well in infection controls. Percentage plaque reduction compared to the infection control was calculated per dilution and plotted as log (inhibitor) vs response curve with four parameter variable slope using GraphPad Prism 9.02. The dilution that would yield 50% reduction of plaques compared to the infection control was estimated by determining the proportionate distance between two dilutions, from which an endpoint titer was calculated. Symbol shapes indicate individual donors and are consistent throughout the figures.

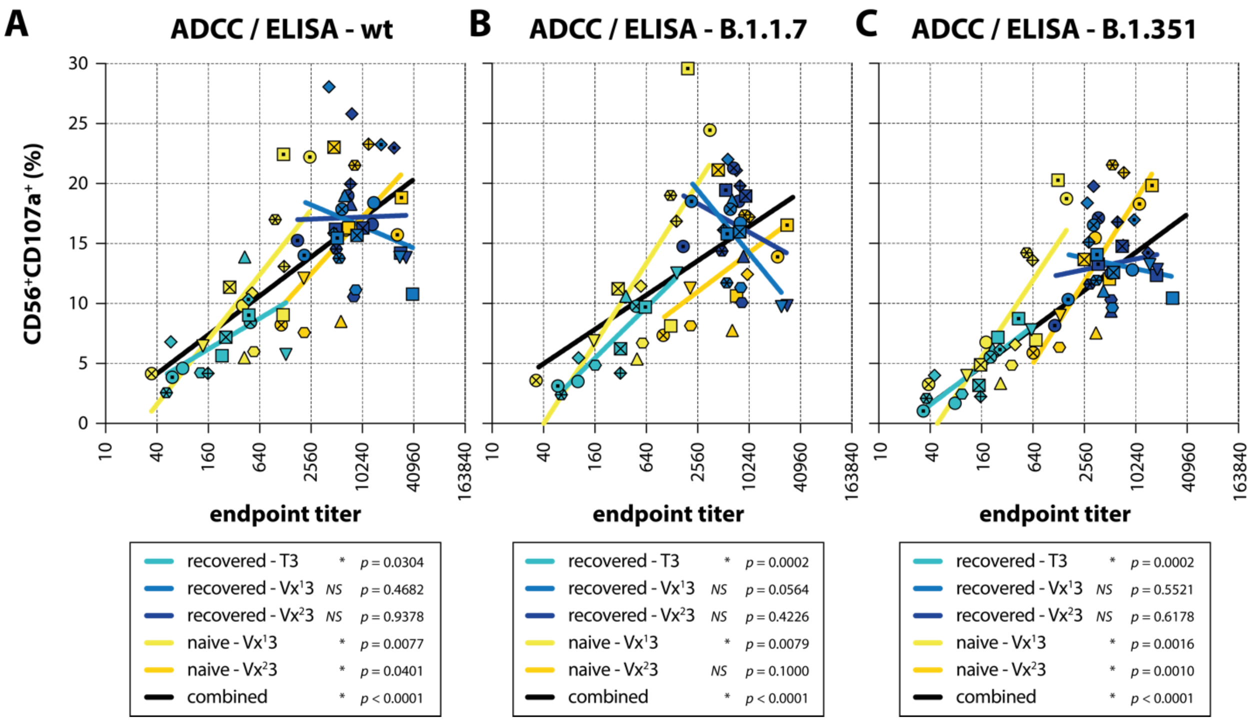

Figure S2. Correlation between antibody binding and Fc-mediated functionality. Direct correlation analyses were performed between antibody binding (**Fig. 2D**) and Fc-mediated functionality (**Fig. 3D**) for (**A**) WT S, (**B**) VOC B.1.1.7 S and (**C**) VOC B.1351 S. Simple linear regression analyses were performed on individual groups / timepoints, and the combination of all timepoints per antigen (black line). *P*-values per regression are indicated below the graphs. Symbol shapes indicate individual donors and are consistent throughout the figures.

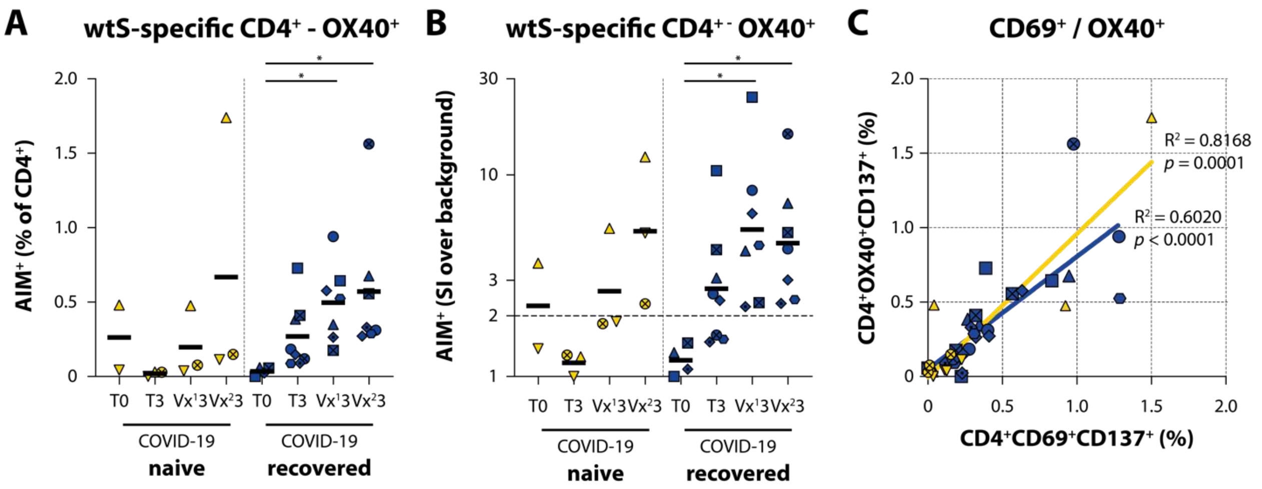

Figure S3. Detection of SARS-CoV-2-specific T cells using OX40. Activation is measured as percentage OX40$^+$/CD137$^+$ double-positive cells within the CD4 fraction, DMSO stimulation is included as background control. (**A, B**) Antigen specific activation of CD4$^+$ T cells in COVID-19 naive (yellow) and COVID-19 recovered (blue) donors at the acute, convalescent, post-vaccination 1 and post-vaccination 2 stage (T0, T3, Vx^{1}3, Vx^{2}3) by overlapping peptide pools covering the full WT S protein. Activation of SARS-CoV-2 specific CD4$^+$ T cells is shown as percentage AIM$^+$ cells within the CD4$^+$ subset after (**A**) subtraction of the DMSO background or (**B**) as a stimulation index (SI) by dividing specific activation over background activation. (**C**) Since OX40 was only assessed for 11 donors, direct correlation analyses were performed between the percentage CD69$^+$/CD137$^+$ CD4$^+$ T cells (**Fig. 4B**) and the percentage OX40$^+$/CD137$^+$ CD4$^+$ T cells. R^2 and *p*-values are indicated in the panel. Timepoints in panel A and B were compared by performing a Kruskal-Wallis test. * $p < 0.05$. Symbol shapes indicate individual donors and are consistent throughout the figures. Lines indicate mean. Low cell count samples (<10,000 events within CD4$^+$ gate) were excluded.

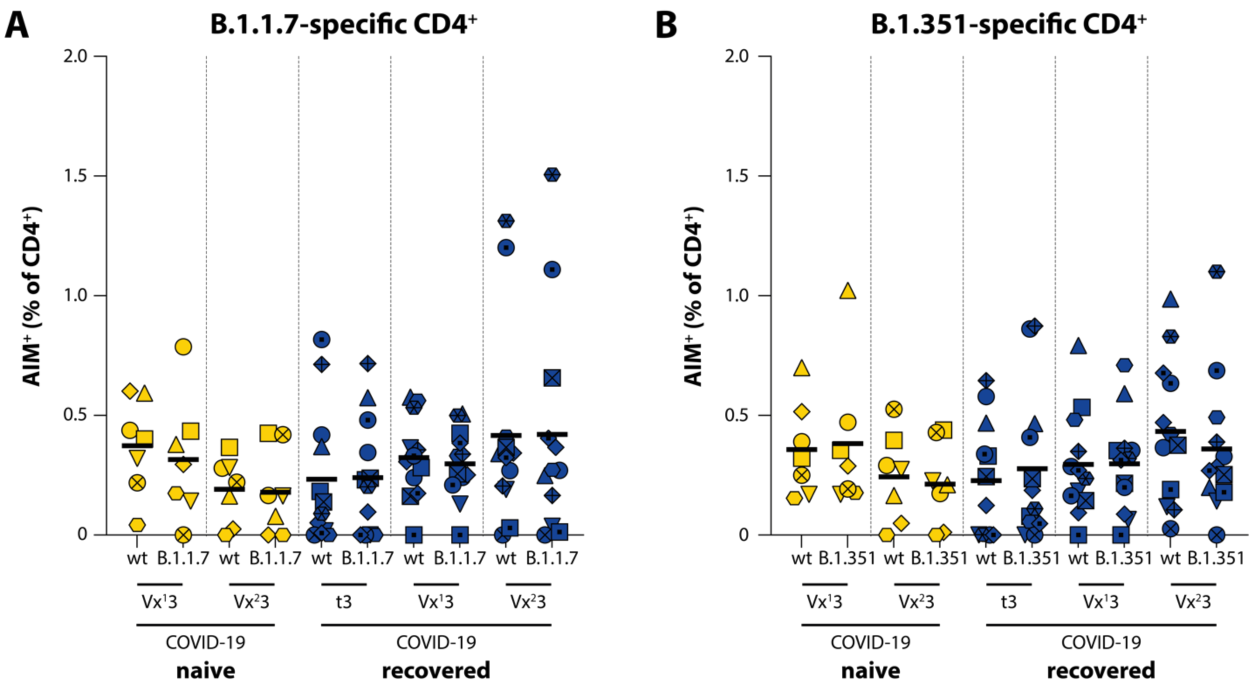

Figure S4. Detection of S-specific T cells by measuring upregulation of activation induced markers (AIM). Activation is measured as percentage $CD69^+/CD137^+$ double-positive cells within the CD4 or CD8 fraction, DMSO stimulation is included as background control. (**A-B**) Antigen-specific activation of $CD4^+$ T cells by peptide pools exclusively covering mutational regions in VOC B.1.1.7 and B.1.351, compared against homologous WT peptide pools. Antigen-specific T-cell responses are shown as percentage AIM positive cells within the $CD4^+$ fraction. These analyses were performed in 20 participants. Symbol shapes indicate individual donors and are consistent throughout the figures. Lines indicate mean responses. Low cell count samples (<10,000 events within $CD4^+$ gate) were excluded.

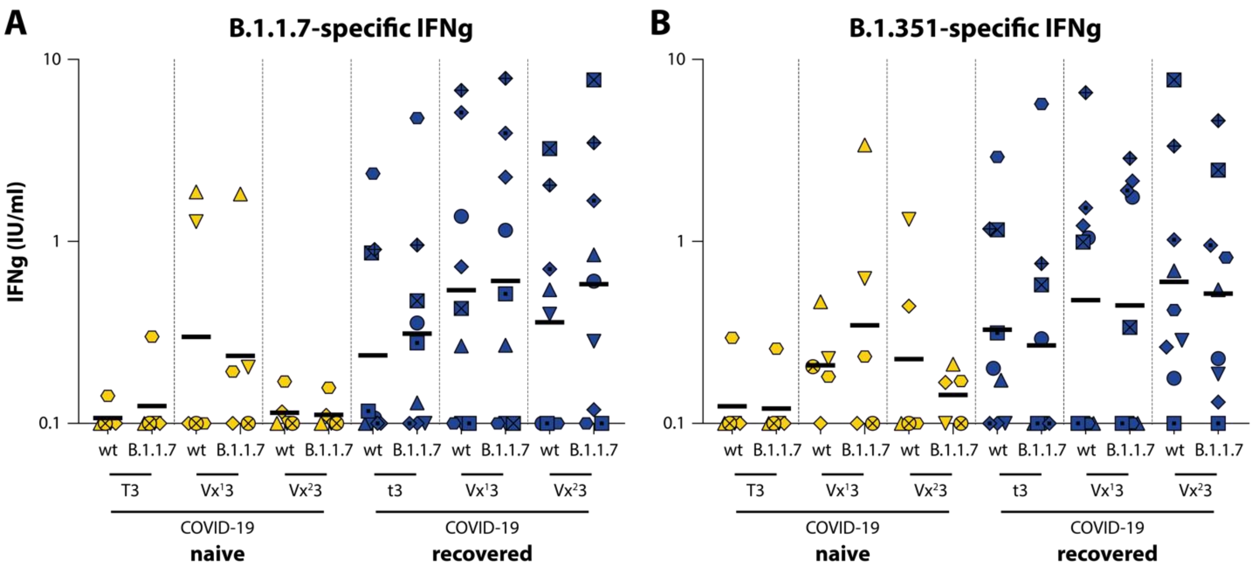

Figure S5. Detection of S-specific T cells by measuring production of IFNγ in culture supernatant. (**A-B**) Antigen-specific activation of $CD4^+$ T cells by peptide pools exclusively covering mutational regions in VOC B.1.1.7 and B.1.351, compared against homologous WT peptide pools. Antigen-specific T-cell responses are shown as IFNγ concentration in the cell culture supernatant and expressed as international units (IU/ml). These analyses were performed in 14 participants. Symbol shapes indicate individual donors and are consistent throughout the figures. Lines indicate geometric mean responses.

Table S1. Overview of immunological responders

	COVID-19 naive (N=12)				COVID-19 recovered (N=13)			
	T0	T3	$Vx^1 3$	$Vx^2 3$	T0	T3	$Vx^1 3$	$Vx^2 3$
**ELISA (N)**	1/12	1/12	1/12	1/12	0/13	11/13	12/13	12/13
>3x background	*8%*	*8%*	*8%*	*8%*	*0%*	*85%*	*92%*	*92%*
**Wantai**	0/12	0/12	10/12	12/12	0/13	13/13	13/13	13/13
>OD450 ratio 1	*0%*	*0%*	*83%*	*100%*	*0%*	*100%*	*100%*	*100%*
**MIA (S1)**	0/12	0/12	12/12	12/12	0/13	13/13	13/13	13/13
>10.08 BAU/ml	*0%*	*0%*	*100%*	*100%*	*0%*	*100%*	*100%*	*100%*
**$PRNT_{50}$**			7/12	12/12		13/13	13/13	13/13
>1:10			*58%*	*100%*		*100%*	*100%*	*100%*
**ADCC (N)**	0/12	0/12	0/12	0/12	0/13	8/13	8/13	6/13
>7% $CD107a^+$	*0%*	*0%*	*0%*	*0%*	*0%*	*62%*	*62%*	*46%*
**ADCC (S)**	0/12	0/12	8/12	12/12	0/13	5/13	13/13	13/13
>7% $CD107a^+$	*0%*	*0%*	*67%*	*100%*	*0%*	*38%*	*100%*	*100%*
**CD4 (S)**	0/5	0/7	5/7	5/7	1/8	8/12	11/13	11/13
>2 SI	*0%*	*0%*	*71%*	*71%*	*13%*	*67%*	*85%*	*85%*
**CD8 (S)**	0/2	0/3	1/7	2/5		1/10	4/12	5/12
>2 SI	*0%*	*0%*	*14%*	*40%*		*10%*	*33%*	*42%*

Table S2: Comparison of geometric mean S1-specific IgG BAU/ml (MIA) between in-depth analyzed sera and all sera

	COVID-19 naive			COVID-19 recovered		
	**T3** (BAU/ml)	**Vx^13** (BAU/ml)	**Vx^23** (BAU/ml)	**T3** (BAU/ml)	**Vx^13** (BAU/ml)	**Vx^23** (BAU/ml)
**In-depth analysis**	1.17	361.82	2854.86	143.16	4820.20	4583.99
**All samples**	1.06	452.10	2982.14	95.84	4748.51	5102.65
*p**	0.34	0.65	0.9	0.42	0.77	0.62

* Groups were compared using Wilcoxon rank test

Table S3. $PRNT_{50}$ fold change reactivity to VOC

		COVID-19 naive			COVID-19 recovered		
		WT	B.1.1.7	B.1.351	WT	B.1.1.7	B.1.351
T3	Geometric mean titer				111.30	319.12	35.68
	95% CI lower limit				72.72	160.24	18.81
	95% CI upper limit				170.34	635.54	71.52
	**Geometric mean ratio**					**+2.86**	**-3.03**
Vx^13	Geometric mean titer	21.37	30.24	12.29	1874.43	4593.30	688.45
	95% CI lower limit	14.24	14.67	9.35	1121.83	2791.83	357.38
	95% CI upper limit	32.06	62.33	9.35	3131.91	7557.22	1326.22
	**Geometric mean ratio**		**+1.42**	**-1.74**		**+2.45**	**-2.73**
Vx^23	Geometric mean titer	189.13	376.15	61.65	2064.53	4486.89	616.73
	95% CI lower limit	106.68	171.73	122.76	1222.53	2916.18	351.74
	95% CI upper limit	335.29	823.90	30.98	3486.45	6903.64	1081.35
	**Geometric mean ratio**		**+1.99**	**-3.07**		**+2.17**	**-3.34**
